# EEG Oscillatory States: Universality, Uniqueness and Specificity across Healthy-Normal, Altered and Pathological Brain Conditions

**DOI:** 10.1371/journal.pone.0087507

**Published:** 2014-02-05

**Authors:** Alexander A. Fingelkurts, Andrew A. Fingelkurts

**Affiliations:** BM-Science – Brain and Mind Technologies Research Centre, Espoo, Finland; University of British Columbia, Canada

## Abstract

For the first time the dynamic repertoires and oscillatory types of local EEG states in 13 diverse conditions (examined over 9 studies) that covered healthy-normal, altered and pathological brain states were quantified within the same methodological and conceptual framework. EEG oscillatory states were assessed by the probability-classification analysis of short-term EEG spectral patterns. The results demonstrated that brain activity consists of a limited repertoire of local EEG states in any of the examined conditions. The size of the state repertoires was associated with changes in cognition and vigilance or neuropsychopathologic conditions. Additionally universal, optional and unique EEG states across 13 diverse conditions were observed. It was demonstrated also that EEG oscillations which constituted EEG states were characteristic for different groups of conditions in accordance to oscillations’ functional significance. The results suggested that (a) there is a limit in the number of local states available to the cortex and many ways in which these local states can rearrange themselves and still produce the same global state and (b) EEG individuality is determined by varying proportions of universal, optional and unique oscillatory states. The results enriched our understanding about dynamic microstructure of EEG-signal.

## Introduction

Electroencephalogram (EEG) still remains one of the main methods (another one is fMRI) in clinical and cognitive neuroscience. It is well known that an EEG has a piecewise stationary structure which is considered to be a result of “gluing” of stationary processes with different probability characteristics (for the reviews see [1]–[7]). It is proposed that each piecewise stationary EEG segment reflects the oscillatory state of a transient neuronal assembly [8]–[15] which signifies a functional cortical *state* [16]–[19]. *State* can be *micro-* or *macro-* depending on finer- or coarser-grained description [20]. The state is *micro* in relation to *macro*state to which it belongs: the important point here is the difference in the amount of detail given by the descriptions. Many different EEG microstates correspond to any one particular macrostate. In such a way, the dynamics of brain activity within a given macrostate can be considered as a sequence of relatively stable brain microstates of different types which are reflected in the EEG as piecewise stationary segments [21]–[23]. Usually microstate is referred to duration of milliseconds and seconds and macrostate corresponds to minutes and hours. An EEG state is a steady, transient and self-organised operational unit [24] which has been proposed to present the basic building blocks of cortical activity accompanied by mentation, thinking and information processing [25]. Activity within each state is stable (or quasi-stable) and is likely to represent a fingerprint of a functionally distinct neuronal network mode. Cortical activity is characterised simultaneously by *local* (specialized) and *global* (integrative) states at each moment in time [4], [8], [19], [26]–[30]. Each local EEG is characterized by sequence of oscillatory states [31]–[33]. Each state from local EEG together with states from other local EEGs at any given moment of time forms a global oscillatory state [34] which is presented as a mosaic of specialized modules constituting nodes in a dynamic network. Each EEG oscillatory state (either local or global) is characterized by multiple EEG oscillations where different oscillations are mixed in different proportions depending on the level of vigilance, perceptual, cognitive and mental operations. Global functional states of the cortex have been extensively studied by means of “momentary brain electric field configurations” ([25], [35]–[37] and others). Local EEG oscillatory states have been examined by means of local short-term power spectra ([31], [38]–[46]; for the review see [47]). The concept of *spectral pattern* (SP) was introduced where the focus is on the overall *shape* of short-term power spectrum rather than on the exact values of the power [48]. For justification for the usage of local short-term SPs for characterisation of local EEG oscillatory states see Appendix S1 (Supporting information).

Intriguingly, these two independent approaches, - one for assessing global functional states and another for examining local functional states of the cortex, - using different methodologies revealed strikingly similar picture: (a) cortex states change in a non-continuous manner: functional state over time shows extended periods during which state is stable (or quasi-stable); these periods of quasi-stability are concatenated by rapid and major changes of state (for global states: [49]; for local states: [31], [40], [45]), (b) a limited number of states exist (for global states: [35], [50]; for local states: [1], [31], [40], [45], [51]), (c) only 4–5 state classes largely dominate the spontaneous EEG in awake, healthy adults (for global states: [50], [52], [53]; for local states: [31]), (d) not all states last equally long and the length of the states is much longer than what one would expect if a random sequence of states is assumed (for global states: [50]; for local states: [31], [45]; [47]), (e) the duration and frequency of appearance of states are altered in different cognitive modes (for global states: [25]; [54]; for local states: [31], [45]) and in several pathological conditions such as depression (for global states: [55]; for local states: [56]) and epilepsy (for global states: [57]; for local states: [34], [58]), as well as after drug intake (for global states: [59]; [60]; for local states: [61], [62]). Such convergence between the results from two independent methodologies suggests that both approaches are reliable and they capture the same temporal principles of neurodynamics but in different spatial scales.

However, several important questions have not yet been answered regarding local EEG oscillatory states. Detailed analysis of the various types of local EEG oscillatory states (in terms of SP types) suggested that an individual (local) EEG across all studied its phenomenological manifestations is best described by a stable and restricted set of SP types [1], [31], [40], [45], [46], [51]. Additionally, the existence of 6 [40] to 11 [45] universal (not necessary dominant) SP types has been demonstrated. These SP types were the same for all studied conditions and it was suggested that they reflect universal short-term quasi-stationary elements which compose the EEG structure [40], [45]. However, to have a more realistic and detailed picture of the number and type of EEG oscillatory states which are *universal*, *unique* or *optional* more studies under more diverse conditions are required. Additionally, several questions need to be answered: (a) Does diversity of local EEG oscillatory states vary as a function of age? (b) Do parameters of dynamic repertoire (that is the set of dynamic behaviours that a neural population can perform in the proximity of its equilibrium state [63]) of local EEG oscillatory states change during cognitive performance, altered states of consciousness and certain neuropsychological disorders? (c) How many SP types are activated by a typical cognitive task or condition, and whether this varies by task category? and (d) How many cognitive tasks or conditions a typical SP supports?

To explore these issues, we performed an aggregated analysis (do not confuse with meta-analysis) of dynamic repertoires of EEG oscillatory states (indexed by SPs types) in 13 conditions (examined over 9 studies), covering healthy-normal, altered and pathological brain states. Aggregated analysis of these previously conducted experimental EEG studies was performed within the same methodological and conceptual framework – the probability-classification analysis of short-term EEG spectral patterns (see EEG data processing below). This methodological approach enables us to reveal peculiarities and generalities of EEG oscillatory states across a multitude of different conditions and tasks which cannot be seen within any one study. Furthermore, such methodological approach establishes whether neuroscientific findings are consistent and can be generalized across populations, settings, conditions or tasks, or whether findings vary significantly by particular subsets. Finally, an aggregated analysis limits bias of individual studies and, hopefully, will improve reliability and accuracy of generalized conclusions.

## Materials and Methods

The aim of this study was to explore the dynamic repertoires and oscillatory types of EEG states in 13 diverse conditions (examined in 9 studies) which cover healthy-normal, altered and pathological brain states. These conditions included: (1) rest with eyes closed in healthy subjects (CE), (2) rest with opened eyes in healthy subjects (OE), (3) waiting period (anticipation of and preparation for an event) of the memory task with opened eyes in healthy subjects (W), (4) memorizing period (information encoding) of the memory task with opened eyes in healthy subjects (M), (5) keeping-in-mind period (information retention) of the memory task with opened eyes in healthy subjects (K), (6) retrieval period of the memory task with opened eyes in healthy subjects (R), (7) benzodiazepine-induced sedation with eyes closed in healthy subjects (B), (8) natural sleep in healthy subjects (S), (9) hypnosis with opened eyes in healthy subject (Hyp), (10) interictal rest with eyes closed in medication-free patients with generalized epilepsy (E), (11) rest with eyes closed in opioid-dependent patients (O), (12) rest with eyes closed in opioid-withdrawal patients (OW), (13) rest with eyes closed in medication-free patients with major depression (D).

The following studies examined the aforementioned conditions: (a) study-1: multistage memory task [45], conditions tested: 1–5; (b) study-2: working memory [64], condition tested: 6; (c) study-3: benzodiazepine sedation [61], condition tested: 7; (d) study-4: major depression [56], conditions tested: 1 and 13; (e) study-5: generalized epilepsy [58], conditions tested: 1 and 10; (f) study-6: opioid dependence [62], conditions tested: 1 and 11; (g) study-7: abstinence [65], condition tested: 12; (h) study-8: hypnosis [66], condition tested: 9; (i) study-9: sleep (not published), condition tested: 8.

All experiments were undertaken with the understanding and written consent of each participant, with the approval of the appropriate local ethics committees, and in compliance with national legislations and the Code of Ethical Principles for Medical Research Involving Human Subjects of the World Medical Association (Declaration of Helsinki) (please see provided references for each study below). Studies 1 and 5 were approved by Moscow State University ethical committee, studies 2, 8 and 9 were approved by University of Turku ethical committee and studies 2, 4, 6 and 7 were approved by Helsinki University Central Hospital ethical committee.

Readers interested in an in-depth discussion and technical details of each of these studies are advised to refer to the provided references. Here we shall briefly describe only some central aspects of each study and characteristics of the computational techniques used.

### 1. Study-1: Multistage Memory Task (See Details in [45])

One-min EEGs were recorded for 12 healthy, right-handed adult subjects (males, aged 19–26) during resting condition (closed and open eyes) and the multistage memory task (waiting, memorizing of the actual matrix object, and retention of the perceptual visual image). Each stage of the memory task was 20-sec in duration.

The visual stimuli presented in front of the subjects to memorize were non-verbalizable matrices composed of nine square elements presented on a matrix screen. The combination of the squares was selected quasi-randomly and presented on the screen for 20-sec by lighting with bottom-mounted red light diodes. Therefore, three distinct short-term (20-sec) periods were tested: before, during, and after the stimulus exposure.

Eight Ag/AgCl electrodes were placed bilaterally on the subject's scalp using the 10/20 system of electrode placement at F_3_, F_4_, C_3_, C_4_, P_3_, P_4_, O_1_, O_2_. Vertical and horizontal electro-oculograms were recorded. All electrodes were referred to linked ears. Raw EEG signals were amplified and filtered in 0.5–30 Hz frequency range and digitized at a sampling rate of 128 Hz by a 12-bit analog-to-digital converter. The impedance of the recording electrodes was always below 5 kΩ. The presence of an adequate EEG signal was determined by visual inspection of the raw signal on the computer screen. All in all 96 (for resting condition with eyes closed) and 97 (for resting condition with open eyes) artifact-free 1-min EEGs and 288 (waiting period of the memory task), 288 (memorizing period of the memory task) and 289 (keeping-in-mind period of the memory task) artifact-free 20-sec EEGs were taken into aggregated analysis.

### 2. Study-2: Working Memory (See Details in [64])

A 20-channel EEG was recorded for 9 healthy, right-handed adult subjects (aged 20–29, 4 males) during a modified Sternberg's memory task. The memory set (encoding) consisted of four auditorily presented stimuli. The frame set (retrieval) size was kept constant and consisted of one stimulus.

The stimuli consisted of 24 auditory verbs (spoken with a female voice). A total of 192 four-verb memory sets were constructed such that each of the verbs had to occur with equal frequency and only once in the same memory set. In 50% of the cases, the frame set verb was among the previously presented four-stimulus block. In total, there were 192 trials, which were presented to the subjects in a pseudorandomized order. The experiment was designed in such a way that it was possible to test separately resting, waiting, encoding, keeping-in-mind, and retrieval short-term periods of the memory task.

In the present study 16 EEG channels (F_7_, F_8_, F_3_, F_4_, F_z_, T_3_, T_4_, C_3_, C_4_, C_z_, T_5_, T_6_, P_3_, P_4_, O_1_, O_2_) were used for the analysis. All electrodes were referred to linked ears. The data were recorded using a sampling rate of 200 Hz with a frequency band of 0.3 to 70 Hz. The impedance of the recording electrodes was always below 5 kΩ. Full EEG streams were split into 5 distinct segments: resting period, waiting period, encoding period, keeping-in-mind period, and retrieval period. A total of 54 artifact-free 1-min EEGs for only retrieval period were taken for the aggregated analysis.

### 3. Study-3: Benzodiazepine Sedation (See Details in [61])

Eight nonsmoking healthy, right-handed human subjects (aged 20–29, 4 males) participated in the study. Participants underwent either lorazepam (Ativan® 4 mg/ml, Wyeth Lederle) 30 µg/kg or placebo (saline) injection in a randomized, double-blind, placebo–controlled crossover design study. The EEG recording began 5 min after the infusion. Two sessions (lorazepam or placebo) were separated by 1 week.

Subjects underwent continuous 10 min (eyes closed and open condition 5 min each) EEG registration with 20 electrodes (F_7_, F_8_, F_z_, F_3_, F_4_, T_3_, T_4_, C_5_, C_6_, C_z_, C_3_, C_4_, T_5_, T_6_, P_z_, P_3_, P_4_, O_z_, O_1_, O_2_) in accordance with the International 10/20 extended system, with a frequency band of 0.06 to 86 Hz and sampling rate of 300 Hz. The impedance of the recording electrodes was always below 5 kΩ and the nose electrode was used as reference. All EEG streams were split into four distinct groups: lorazepam–eyes-closed, lorazepam–eyes-open, placebo–eyes-closed, placebo–eyes-open. A total of 40 artifact-free 1-min EEGs for only lorazepam–eyes-closed were taken for the aggregated analysis.

### 4. Study-4: Major Depression (See Details in [56])

Twelve medication-free outpatients with depression (mean age 43.5±13.3 years, all right-handed, 7 males) and ten sex- and age-matched non-smoking healthy controls (mean age 40±12.9 years, all right-handed, 5 males) participated in the study. All subjects underwent a Structured Diagnostic Interview (SCID) for DSM-III-R. All outpatients with depression met the DSM-III-R criteria for a major depressive episode. They also had a score of at least 18 on the 17-item Hamilton Depression Rating Scale (HAM) at the time of the study procedure (the group mean HAM score was 23.7±4.2). All controls were free from psychiatric illnesses and the mean HAM score for the control group was 0.5±0.8.

Subjects underwent EEG registration in accordance to the International 10/20 extended system, 20 minutes in duration during eyes closed rest with a frequency band 0.06–86 Hz and sampling rate of 300 Hz. The impedance of the recording electrodes was always below 5 kΩ and the nose electrode was used as reference. In the present study EEGs from 20 electrodes (F_7_, F_8_, F_z_, F_3_, F_4_, T_3_, T_4_, C_5_, C_6_, C_z_, C_3_, C_4_, T_5_, T_6_, P_z_, P_3_, P_4_, O_z_, O_1_, O_2_) were analyzed. EEG data were split into two distinct groups: “depressive” and “control.” A total of 182 (for eyes closed rest in healthy subjects) and 206 (for eyes closed rest in medication-free patients with major depression) artifact-free 1-min EEGs were taken for the aggregated analysis.

### 5. Study-5: Generalized Epilepsy (See Details in [58])

Six medication-free right-handed patients with generalized epilepsy (aged 17–40, 3 females) were selected for the study. Inclusion criteria were the persistent presence of epilepsy for more than one year, and the absence of (a) any epileptiform activity in the interictal EEG (that refers to the period of time between seizures), and (b) any neurological condition other than epilepsy, or any acute or chronic medical illness at the time of the EEG registration. Interictal epileptiform activity was identified via visual inspection according to the criteria laid down by the International Federation of Societies for Electroencephalography and Clinical Neurophysiology. All patients were in good physical health, determined by a physical examination and laboratory evaluation including a complete blood count, glucose, and hepatic enzymes, renal and thyroid analyses. Patients could have taken medication for extended periods but not during the final two weeks before EEG registration.

Seven sex- and age-matched healthy control subjects (aged 19–35, 3 females) participated in the study. Before inclusion, the control subjects underwent a medical examination and were also screened for EEG epileptiform activity. All control subjects had epileptiform-free EEGs.

Five 1-min EEGs were recorded for each subject during resting condition (closed eyes). Sixteen Ag/AgCl electrodes were placed bilaterally on the subject's scalp using the 10/20 system of electrode placement at O_1_, O_2_, P_3_, P_4_, C_3_, C_4_, C_z_, T_3_, T_4_, T_5_, T_6_, F_3_, F_4_, F_z_, F_7_, F_8_. Vertical and horizontal electro-oculograms were recorded. All electrodes were referred to linked ears. Raw EEG signals were amplified and bandpass-filtered in the 0.5–30 Hz frequency range and digitized at a sampling rate of 128 Hz by a 12-bit analog-to-digital converter. The impedance of the recording electrodes was always below 5 kΩ. A total of 18 (for interictal condition in epileptics) and 14 (for resting condition in control subjects) artifact-free 1-min EEGs were taken for the aggregated analysis.

### 6. Study-6: Opioid Dependence (See Details in [62])

The study included a total of 22 right-handed opioid-dependent patients (aged between 21 and 46 years, 14 males) and 14 right-handed controls (mean age 33.3±6.4 years, 6 males). All patients had abused opioids for 4–26 years (mean 11 years). Self-reported daily dose was 0.05–2 g for intravenous administration of heroin and 2–32 mg for intravenous administration of buprenorphine. All 22 patients met DSM-IV criteria for opioid dependence, while healthy controls did not fulfill any criteria for DSM-IV disorders on Structured Clinical Interviews I and II. Neuropsychologic tests showed normal intelligence in all subjects.

The patients were investigated on the day of admission, and all had abused opioids within 12 h before EEG registration; the dosages were the patients' usual dosages. None of the patients had a withdrawal syndrome at the time of the EEG registration, as verified by a Gossop test. Each subject underwent 5 min of EEG registration during eyes closed rest with a frequency band of 0.06 to 86 Hz and sampling rate of 600 Hz. The impedance of the recording electrodes was always below 5 kΩ and the nose electrode was used as reference.

In the present study EEGs from 20 electrodes (F_7_, F_8_, F_z_, F_3_, F_4_, T_3_, T_4_, C_5_, C_6_, C_z_, C_3_, C_4_, T_5_, T_6_, P_z_, P_3_, P_4_, O_z_, O_1_, O_2_) were analyzed. EEG data were split into two distinct groups: “opioid” and “control.” A total of 110 (for opioid dependence condition) and 70 (for resting condition in control subjects) artifact-free 1-min EEGs were taken for the aggregated analysis.

### 7. Study-7: Abstinence (See Details in [65])

In the study 13 right-handed, opioid-dependent patients (mean age 32±5 years, 8 males) and 14 controls (mean age 33.3±6.4 years, 6 males) participated. All patients had abused opioids for 4–26 years (mean 10 years). Self-reported daily dose was 0.05–1.2 g for intravenous administration of street heroin and 2–16 mg for intravenous administration of street buprenorphine. All patients met DSM-IV criteria for opioid dependence, while all controls did not fulfill any criteria for any DSM-IV disorder.

At the time of the EEG assessment, patients had been abstinent for 12–15 days. The severity of withdrawal syndrome was verified by Gossop test. Each subject underwent 5 min of EEG registration during eyes closed rest with a frequency band of 0.06–86 Hz and sampling rate of 600 Hz. The impedance of the recording electrodes was always below 5 kΩ and the nose electrode was used as reference.

In the present study EEGs from 20 electrodes (F_7_, F_8_, F_z_, F_3_, F_4_, T_3_, T_4_, C_5_, C_6_, C_z_, C_3_, C_4_, T_5_, T_6_, P_z_, P_3_, P_4_, O_z_, O_1_, O_2_) were analyzed. EEG data were split into two distinct groups: “withdrawal” and “control.” A total of 65 artifact-free 1-min EEGs for opioid-withdrawal condition were taken for the aggregated analysis.

### 8. Study-8: Hypnosis (See Details in [66])

The subject, a 39-year-old right-handed female without a history of neurological or psychiatric illness was an experienced participant in hypnosis experiments. The participant displayed all phenomena typically associated with very highly susceptible individuals (virtuosos), such as vivid hallucinations and amnesia. Furthermore the participant instantly responded to a posthypnotic suggestion (e.g. a word or a sign) about “entering hypnosis” which made it possible to induce or terminate hypnosis without standard experimental induction procedures.

The subject had previously been given a posthypnotic suggestion about entering hypnosis or waking when hearing the experimenter say certain pseudowords. During the experiment, hypnosis was induced and terminated with this technique. The only instruction given to the subject was to focus on a LED-light in front of her (about 2 meters distance) and avoid unnecessary eye movements. Each session started with two minutes of EEG data acquisition (baseline condition) while the subject sat in a comfortable chair and had her eyes open and focused on the LED-light. This was followed by three EEG acquisition blocks where hypnosis and non-hypnosis (being induced by a posthypnotic suggestion) followed each other. Each block consisted of 3–4 hypnosis and non-hypnosis periods, each lasting about 2 minutes. The three blocks (each lasting about 10 minutes) were separated by a break of about 5 minutes in normal waking state while the subject could stretch herself. The sequence of hypnotic and non-hypnotic conditions was varied so that each condition started the blocks equally often. The hypnosis and non-hypnosis periods within the blocks also varied (+/−30 seconds) in order to prevent the subject from anticipating the change.

Spontaneous electrical brain activity was recorded with a 20 EEG channels (Fp_1_, Fp_2_, F_7_, F_8_, F_Z_, F_3_, F_4_, T_3_, T_4_, C_3_, C_4_, C_Z_, T_5_, T_6_, P_Z_, P_3_, P_4_, O_Z_, O_1_, O_2_) with a frequency band of 0.05 to 100 Hz (sampling rate of 500 Hz). EEG was recorded with an electrode cap in accordance to the International 10/20 extended system; the nose electrode was used as reference. The impedance of each electrode was monitored before data acquisition and was always below 5 kΩ. Vertical and horizontal electro-oculograms were recorded. A total of 22 artifact-free 1-min EEGs for hypnotic condition were taken for the aggregated analysis.

### 9. Study-9: Sleep (Not Published)

Nine subjects (mean age 23.7±1.51 years, 4 females) participated in the study. Six 1-min EEGs were recorded during Stage 2 and Stage 3 of NREM sleep during the first half of the night. The impedance of the recording electrodes was always below 5 kΩ and right ear mastoid was used as reference. In the present study EEGs from 20 electrodes (Fp_1_, Fp_2_, F_7_, F_8_, F_z_, F_3_, F_4_, T_3_, T_4_, C_z_, C_3_, C_4_, T_5_, T_6_, P_z_, P_3_, P_4_, O_z_, O_1_, O_2_ in accordance to the International 10/20 extended system) were analyzed. A total of 27 artifact-free 1-min EEGs for sleep condition were taken for the aggregated analysis.

### 10. EEG Artifacts Control

EEG epochs containing artifacts (amplitude >80 µV) due to eye blinks, significant muscle activity, and movements were automatically removed. In two studies (study-6 and study-7) EEG components containing artifacts were automatically removed by means of ICA (Independent Component Analysis) procedure. The presence of an adequate signal was determined by visually checking each raw signal on the computer screen after automatic artifact rejection. If some residual artifacts were still present they were marked and then automatically rejected from further analysis.

Instructions designed to minimize movement and relax jaw muscles resulted in suppressing the myogram class of artifact to the extent that the high-frequency spectrum was not significantly affected. Cardiac interference at low frequencies was also found to be minimal, with no spectral peak-detection at the heartbeat frequency of around 1 Hz, or its harmonics. The subjects from all studies were instructed also to look straight in front of them (even when the eyes were closed) and to avoid unnecessary eye movements.

### 11. EEG Data Processing

The basic procedure for data analysis was as follows: for each subject a full EEG stream free from any artifacts was fragmented into consecutive 1-minute epochs (or 20-sec epochs for study-1). This permitted us (a) to normalize EEG data from different studies for the length (in different studies EEG duration was different): EEG oscillatory states were quantified for the period of 1-minute (or 20-sec) and (b) to obtain a relatively large number of analyzed epochs for each subject. All 1-minute (or 20-sec) EEGs were split into groups which corresponded to the aforementioned conditions within each study. Within each group further data processing was performed for each separate 1-minute (or 20-sec) portion of the signal. Due to the technical requirements of the tools used to process the data, EEGs were re-sampled to 128 Hz in cases where the raw EEG was recorded at a higher sampling rate. This procedure should not affect the results since 128 Hz sampling rate meets the Nyquist Criterion [67] of a sample rate greater than twice the maximum input frequency which is sufficient to avoid aliasing and preserve all the input signal information.

**Figure 1:**
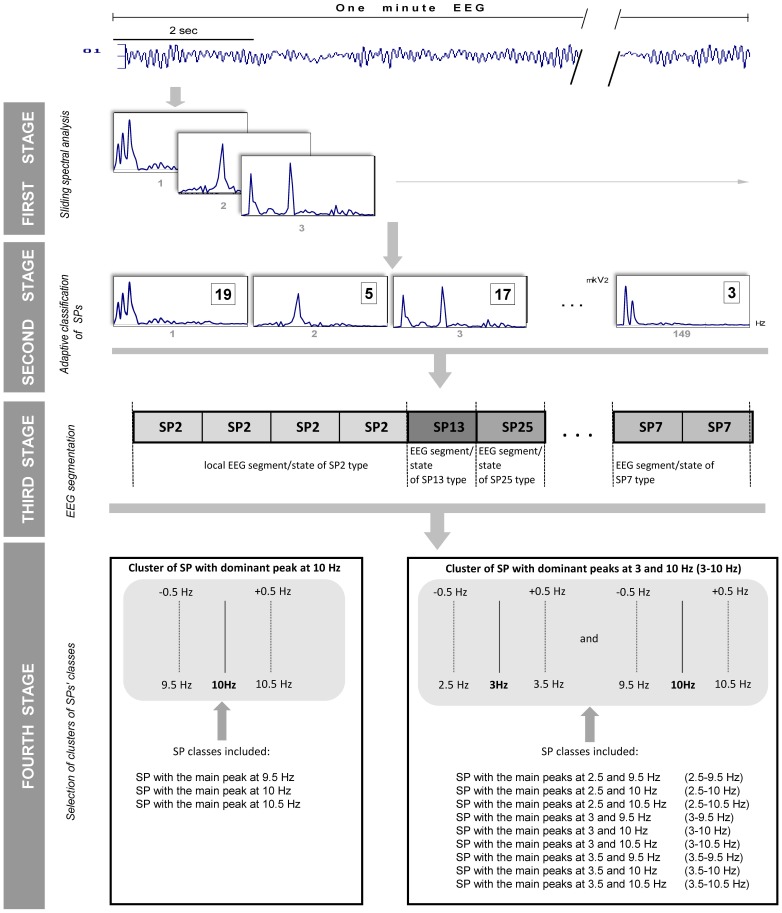
Data processing scheme. *First stage*: Sliding spectral analysis was conducted separately for each subject and each channel of 1-minute (or 20-sec) EEG. O1 = Left occipital EEG channel. *Second stage:* Adaptive classification of short-term spectral patterns (SP) was performed separately for each subject and each channel of 1-minute (or 20-sec) EEG. The small gray numbers under each SP represent the running numbers from 1 to 149 for 1-minute EEG. The number in the square represents the class to which a given SP was assigned to during the classification procedure. *Third stage*: Segmentation of the EEG signal was performed based on SP type changes for each EEG channel separately. The moment of SP-segment type change marks an accompanying change in EEG oscillatory state. *Fourth stage*: Selection of clusters of SP’s classes based on natural variability of resonance frequencies within ±0.5 Hz.

After re-sampling, local EEG oscillatory states were identified for each EEG channel separately: this procedure was undertaken in four stages (Fig. 1). During the first stage of EEG analysis data series from each EEG channel were divided into overlapping windows in order to capture changing EEG dynamics. Local EEG oscillations were quantified by calculation of individual short-term EEG SPs. Individual power spectra were calculated in the range of 1–30 Hz with 0.5-Hz resolution, using Fast Fourier Transform with a 2-sec Hanning window shifted by 50 samples (0.39-sec) for each one-minute EEG channel (Fig. 1). The uniformity of the time window across frequencies is considered a limitation of FFT [68], [69]. However, chosen frequency range of 1–30 Hz and parameters for FFT (2-sec Hanning window and 50 samples lag) have proved to be the most effective for extracting oscillatory patterns from the signal based on previous studies [70]–[73]. Sliding spectral analysis with overlapping segments, previously applied to EEG signals [74], [75], (a) takes the non-stationarity of the time series into account, (b) compensates for the effects of windowing, (c) detects more clearly systematic oscillatory changes in the signal’s activity and (d) prevents loss of information due to residual activity. Additionally, using overlapping intervals (which just means a different aggregation scheme) cannot add any artefactual information [76]. Effects of sliding analysis on SP types can be found in [45].

Log transformation of the power spectra was not used in the present study for the following reasons: Log transformation usually normalizes a power spectrum, but, at the same time, it artificially reduces the contrast of the differences between large and small power values. This leads to an increased contribution of the small amplitude values and correspondently the noise into a total spectrum. For the purpose of this paper “clean” power spectra without noise contamination are of great importance. Additionally, log transformation can exaggerate extremely small, but topographically reproducible errors in areas with low EEG power.

1–30 Hz frequency range was chosen because approximately 98% of spectral power lies within these limits [77]. Although it has recently been proposed that frequencies above 30 Hz (gamma band) may be functionally informative, there are a number of methodological issues which lead us to exclude frequencies above 30 Hz from the analysis: (a) it was shown that volume conduction has little influence on the shape of the spectrum below around 25 Hz, however spatial filtering is significant for frequencies above 25 Hz [78]; (b) high-frequency spindles have a very low signal-to-noise ratio, which results in considerable noise contamination of the gamma band; (c) the dynamics of high-frequency effects may be a trivial by-product of power changes in lower frequencies [79], (d) increased power in the gamma range may be due to the harmonics of activity in lower frequency ranges, and/or due to the ringing of filters by EEG spikes recurring at theta rates [33], (e) the gamma band may be an artefact of (un)conscious micro-constrictions of body and/or face muscles [80]–[82]; (f) comprising just 2% of the spectral power [77], contribution of high-frequency band to the spectrum cannot be significant; (g) Bullock et al. [83] demonstrated many “good” rhythms in the 2–25 Hz range which were mainly sinusoidal but did not find them in the 30–50 Hz band. In the light of the above, there may be difficulties in carrying out a meaningful interpretation of effects at the high-frequency band regardless of how powerful or statistically significant they are.

Further, interpretations of gamma band in relation to higher cognitive activity are over-emphasized since: (a) isolated ganglia of invertebrates also show significant gamma responsiveness [84] and (b) gamma oscillations are present during states such as sleep, deep anesthesia and persistent vegetative state, where conscious cognitive processing is absent [85]–[88].

DC drifts were removed using high pass filters (1 Hz cut-off).

After the calculation of short-term EEG SPs, the total number of individual SPs for each channel of 1-minute EEG was 149 (Fig. 1) and 50 for each channel 20-sec EEG. The number of 1-minute (or 20-sec) EEGs available for each subject varied between 5 and 24 depending on study and condition.

During the second stage, each SP was labelled according to the class index it belongs to, with the help of a probability-classification analysis of the short-term EEG SPs (published in [45], see also Appendix in [89]). A probability-classification analysis was performed automatically in four steps separately for each channel of 1-minute (or 20-sec) EEG. During *first step* a set of *standard* SPs was generated automatically: a pool of SPs was built from all the SPs of the entire EEG signals (all locations) for all subjects within each study (*N_pool_*_ = _149 (or 50) SPs×*n* EEG channels×*n* one-minute (or 20-sec) EEGs×*n* subjects). From this pool (*N_pool_* = 14 016–3 410 968 of SPs depending on the study), all identical SPs with dominant power peaks (peaks that rise significantly above the general average) were counted automatically. The peak detection was based on normalizing the SP to within-SP relative percentages of magnitude, where acceptance is achieved when the peak exceeds a given (60%) percent-magnitude (100% corresponds to the magnitude of the highest peak within the SP). According to the preliminary study, this value has proved to be the most effective for peak detection. The set of SPs with the highest count were the most probable candidates to form the “set of standard SPs.” Only those SPs with a minimal mutual correlation were selected for standard SPs set (first step). Notice that there is no universal set of standard SPs: each EEG data (different studies) require formation of new set of standard SPs. According to our experience the sets of standard SPs from different studies overlap significantly, but they are not identical neither in number of SPs, nor in SP’s types.

During the *second step*, the initial matrix of cross-correlations (Pearson’s correlation coefficients, CC) between standard and current individual SPs of analyzed EEG was calculated for each channel separately. The current SPs that their CC passed the acceptance criteria of *r* ≥0.71 were attributed to their respective standard classes. Therefore, the same current SPs may be included simultaneously into different standard classes. The CC acceptance criteria *r* was determined such as for *r* ≥0.71 more than 50% of the SP variances were coupled/associated.

During the *third step*, the current SPs included in a particular class were averaged within this class. The same procedure was performed for all classes separately for each EEG channel. On the back of this, the standard spectra were reconstructed but this time taking into account the peculiarities of the spectral description of concrete channel of the particular EEG. In this way an “actualization” of the initial standard SP set was performed. In other words, standard SPs were converted into so-called *actual* spectral patterns. Notice that the main frequency peaks in the *actual* SP of every class stay the same as in the corresponding *standard* SP’s classes. However, overall shape of the power spectrum was automatically modulated in the direction to better represent the multitude of all SPs within each class in each given EEG channel.

An actual SP set was in turn used for the *fourth step*–the final classification of the current SPs: each of current SPs was attributed to only one actual SP class for which the CC was the *maximum* of the set of *r* ≥0.71.

The probability-classification technique employs correction algorithm to achieve a significant reduction in the variance of single spectral estimations and to take into account the relationship between neighbour frequencies in the frequency continuum: choosing the maximum CC out of the three values of the correlation function, which was calculated between the *standard* SP and the *current* SP on zero shift and on double-side shift by one step (±0.5 Hz). This increases the sensitivity of this analytical approach in revealing the dynamics of EEG oscillatory patterns. Described SP classification method made it possible to identify up to 100% of the individual single spectra in the EEGs due to the algorithm’s ability to adapt to local signals. Therefore at every time step a valid classification was reached, i.e., there was no ‘undecided’ category.

As a result of the probability-classification procedure, each current SP was labelled according to the index of the class to which it belonged. Hence, each EEG signal was reduced to a sequence of individually classified SPs. Thus, a sequence of SP labels that represents the sequence of EEG oscillatory states through which the system passes was obtained (Fig. 1). Examples of SP types can be found in our previously published papers (see Fig. 3 in [64]; Fig. 1 in [46], [56], [58], [66]; Fig. 7 in [89]; and Fig. 2 in [113]).

**Figure 2:**
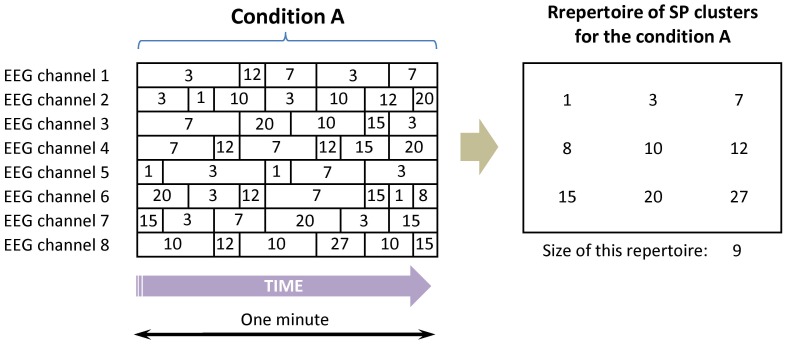
Schematic representation of the formation of repertoire of SP clusters for a given condition. The type (labelled by the number), duration and the number of SP clusters for each EEG channel are presented. Relative frequency of each SP cluster occurrence was averaged separately for each EEG channel across all EEGs and all subjects for a given condition. The repertoire of SP clusters for this condition is a multitude of the types of SP clusters occurred during a given condition. If an SP cluster was found in one or more EEG channels for all EEGs (in average) for a given condition within a given study then this type of SP cluster was assigned to the repertoire of that particular condition.

**Figure 3:**
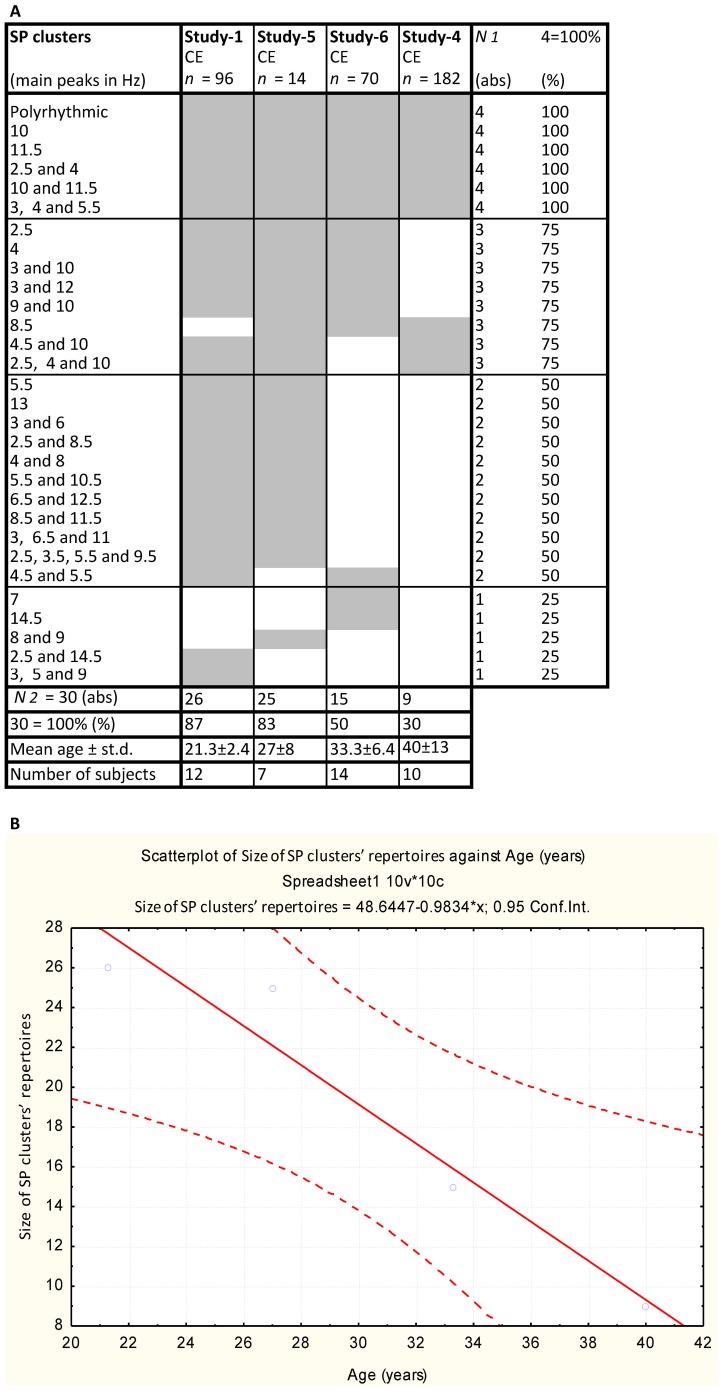
Repertoires of SP clusters in four resting conditions with eyes closed (examined over four studies). (**A**). Only healthy subjects from each study were taken into analysis. A sequence of four studies arranged in accordance to the mean age of subjects participated from younger to older age. The list of SP clusters is organised in accordance with the frequency of each SP cluster occurrence across 4 resting conditions, the most frequent being on the top. CE = closed eyes, *n* = the number of EEGs for each condition, *N_1_* = the number of studies for which a given SP cluster has been observed, *N_2_* = the number of SP clusters in repertoires for each condition, abs = absolute values, st.d. = standard deviation. (**B**) Scatter plot of size of SP clusters’ repertoires vs. age is presented.

During the third stage, each EEG channel was segmented (Fig. 1) based on SP type changes. A single EEG spectrum reflects the coordinated work of tens and hundreds of thousands of neurons at a particular point in time [90]. Therefore, the absence of variance of a single SP type during several analysed epochs suggests that the same macro-regimen of neuronal pool activity is maintained throughout that period. Thus, periods of several consecutive EEG epochs which are characterized by the same SP type comprise an SP-segment i.e. an EEG segment of quasi-stationary oscillatory activity or an EEG oscillatory state. The moment of change from one type of SP-segment to another marks a transition in EEG oscillatory state (see Fig. 1) within each EEG channel. Therefore the duration of states varies (Fig. 1, Third Stage). Temporal coordinate of boundary of a given SP-segment is dependent on a discrete temporal lag of 0.39 s used for calculation of SPs (see above). Considering that the shift in 0.39 s was the most effective on disclosing oscillatory patterns from the signal in modelling study [73], one may assume that measured temporal coordinates of boundaries of SP-segments approach the real ones (in frequency domain). In such a way the types of EEG oscillatory states (indexed by SP types) and their number were obtained (Fig. 1) for each EEG channel and studied condition.

It is known that peak frequency of characteristic EEG oscillations is fairly stable and in most individuals varies about 0.5 Hz from day to day or during a single session [91]–[93] and it is also consistent for various subject populations [94], [95]. In order to accommodate this natural variability in peak frequency, SP classes for each EEG channel within each study were grouped into clusters during the fourth stage as shown in Figure 1. If an SP cluster was found in one or more EEG channels for all EEGs (in average) for a given condition within a given study then this type of SP cluster was assigned to the repertoire of that particular condition (Fig. 2). This means that a certain SP cluster should appear at least in one EEG channel in the vast majority (≥80%) of subjects for a particular condition, to be included in the repertoire of that particular condition. Therefore a given repertoire of SP clusters for a given condition represents a multitude, where different SP clusters come from the same or different channels of 1-minute (or 20-sec) EEG (Fig. 2). Parameters of dynamic repertoires of SP clusters were compared across 13 conditions and 9 studies.

### 12. Statistics

Correlations between variables were assessed by Spearman correlations test. Differences in the percent changes between reference state (resting condition with closed eyes) and other conditions were assessed by Chi-square test. Statistical significance was assumed where *p*<.05.

## Results

To study the size, typicality and uniqueness of dynamic repertoires of SP clusters and the chance likelihood of occurrence for each SP cluster across normal and various pathological brain conditions, we will contrast these conditions with a common reference state – *resting condition with closed eyes in healthy subjects*, which is usually defined as a “*baseline*” of brain activity.

### 1. Parameters of Dynamic Repertoire of SP Clusters and the Frequency of each SP Cluster Occurrence during Resting Condition with Closed Eyes in Healthy Subjects

Figure 3 summarises dynamic repertoires of SP clusters for resting condition with closed eyes examined in four independent studies (only healthy subjects from each study were taken in the analysis). It can be seen that the resting EEG in each study was characterised by a limited repertoire of SP clusters which ranged from 9 to 26 types. Note that the number of SP clusters in the resting condition correlated negatively with age: the higher the number of SP clusters in the repertoire, the younger the mean age of subjects (*r* = −0.96, *p*<.05, Spearman correlations; Fig. 3).

All four repertoires overlapped significantly: only 1 or 2 SP clusters were unique in the three studies (studies- 1, 5 and 6); repertoire of SP clusters in study-4 completely overlapped with the other three repertoires (Fig. 3). Notice that there was no correlation between the number of EEG channels and the number of SP clusters in the repertoire (the size of repertoire).

When all four repertoires were taken together, the size (number) of the overlapping repertoire of SP clusters reached 30 (Fig. 3). Analysis revealed that almost half (47%) of SP clusters from this common repertoire occurred in any three or all four studies, 37% of SP clusters from the common repertoire occurred in any two studies and only 16% of the SP clusters occurred in any one study.

Thus, this common repertoire is characteristic for all examined manifestations of closed eyes resting condition EEG in a wide age range (from 19 to 60 years) in healthy subjects and will serve us as baseline reference of EEG oscillatory activity.

### 2. Parameters of Dynamic Repertoires of SP Clusters and the Frequency of Each SP Cluster Occurrence Across Diverse Healthy and Pathological Conditions

#### 2.1. Size (number) of the repertoires of SP clusters

**Figure 4:**
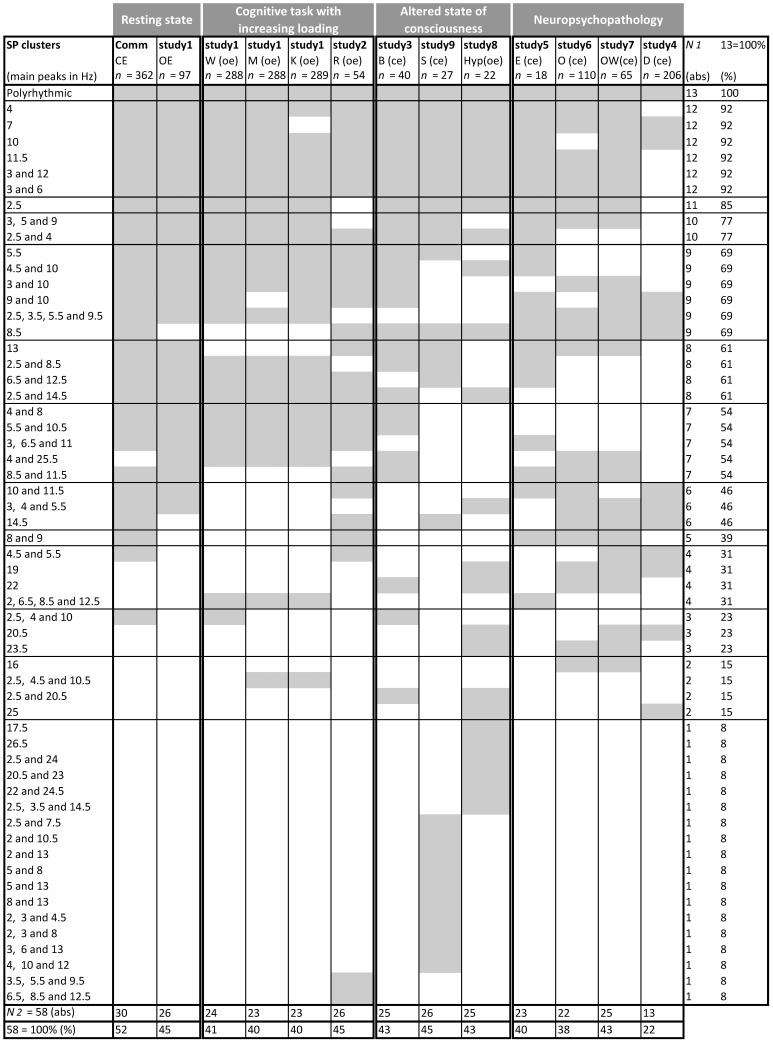
Repertoires of SP clusters for 13 diverse conditions (examined over 9 studies). The sequence of conditions was arranged in four groups: resting conditions, cognitive task with increased loading, altered states of consciousness, and neuropsychopathology. The list of SP clusters is organised according to the frequency of each SP cluster occurrence across 13 conditions, the most frequent being on the top. CE = closed eyes, OE = open eyes, Comm = common for four resting conditions in healthy subjects, W = waiting period of the memory task in healthy subjects, M = memorizing period of the memory task in healthy subjects, K = keeping-in-mind period of the memory task in healthy subjects, R = retrieval period of the memory task in healthy subjects, B = benzodiazepine-induced sedation in healthy subjects, S = natural sleep in healthy subjects, Hyp = hypnosis in healthy subject, E = interictal rest in medication-free patients with generalized epilepsy, O = rest in opioid-dependent patients, OW = rest in opioid-withdrawal patients, D = rest in medication-free patients with major depression, *n* = the number of EEGs for each condition, *N_1_* = the number of conditions for which a given SP cluster has been observed, *N_2_* = the number of SP clusters in repertoires for each condition, abs = absolute values.

Figure 4 summarises dynamic repertoires of SP clusters for 13 diverse healthy and pathological conditions: rest with closed eyes (CE), rest with opened eyes (OE), waiting stage of the memory task (W), memorising stage of the memory task (M), keeping-in-mind stage of the memory task (K), retrieval stage of the memory task (R), benzodiazepine-induced sedation with closed eyes (B), natural sleep (S), hypnotic rest with opened eyes (Hyp), interictal rest with closed eyes in patients with generalised epilepsy (E), opioid addiction rest with closed eyes (O), opioid withdrawal rest with closed eyes (OW), rest with closed eyes in patients with major depression (D).

**Figure 5:**
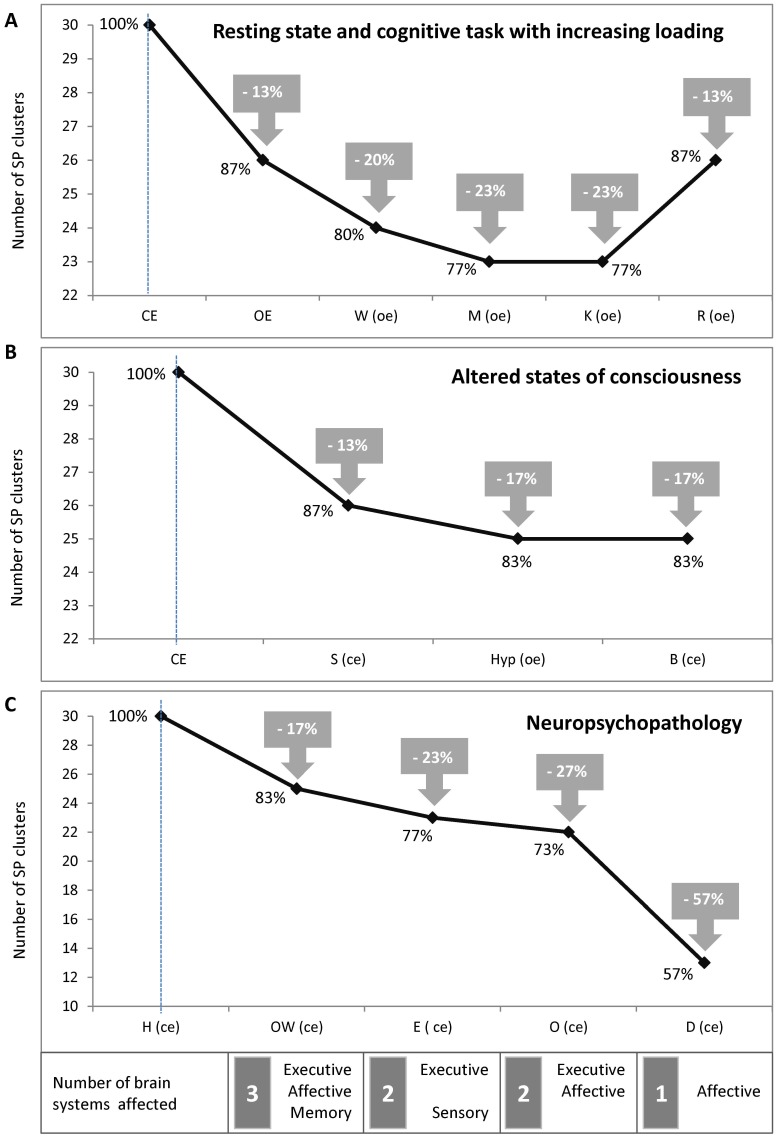
Repertoires size of SP clusters for different conditions. The closed eyes rest condition in healthy subjects is taken as a reference functional state and it is indicated on the figure as a vertical line. The sequence of conditions is arranged in accordance to (A) cognitive loading, the maximum loading being on the right; (B) the level of consciousness alteration, the maximum alteration being on the left; and (C) the number of the predominantly impaired brain systems (according to [96]), the maximum number being on the left. CE = closed eyes, OE = open eyes, W = waiting period of the memory task in healthy subjects, M = memorizing period of the memory task in healthy subjects, K = keeping-in-mind period of the memory task in healthy subjects, R = retrieval period of the memory task in healthy subjects, S = natural sleep in healthy subjects, Hyp = hypnosis in healthy subject, B = benzodiazepine-induced sedation in healthy subjects, OW = rest in opioid-withdrawal patients, E = interictal rest in medication-free patients with generalized epilepsy, O = rest in opioid-dependent patients, D = rest in medication-free patients with major depression.

It can be seen that the EEG of each condition was characterised by limited repertoire of SP clusters which ranged from 13 to 30 types (Fig. 4). The largest repertoire of SP clusters was observed for resting condition with closed eyes. Progressive increase in cognitive loading resulted in considerable narrowing (by 13–23%, *p*<.001) of correspondent repertoires, reaching the minimum during memorisation (M) and retention (K) of information (Fig. 5, A). Additionally, natural (sleep) or induced by hypnosis or benzodiazepine alteration of consciousness also resulted in repertoires’ reduction (by 13–17%, *p*<.02, Fig. 5, B). Finally, all examined neuropsychopathologic resting conditions demonstrated considerably smaller repertoires of SP clusters (by 17–43%, *p*<.0000001) when compared with healthy resting condition (Fig. 5, C). The smallest repertoire of SP clusters was observed in the depressive condition. Omitting depressive condition still holds significant result: reduction of repertoires of SP clusters was by 17–27% (*p*<.0004). Note that the more brain systems were predominantly impaired (according to [96]) the bigger was the size of repertoire of SP clusters in neuropsychopathologic conditions (*r* = - 0.95, *p*<.05, Spearman correlations; Fig. 5, C).

When compared to resting closed eyes condition the largest decrease in the size of repertoires of SP clusters was observed during neuropsychopathologic conditions, moderate decrease was seen during cognitive loading and smallest for conditions of altered states of consciousness (Fig. 5).

#### 2.2. Uniqueness/universality of SP clusters

Analysis of the frequency of each SP cluster occurrence across 13 diverse conditions covering healthy and pathological states revealed that (a) 8 SP clusters were *universal* (observed in more than 11 (≥85%) conditions), (b) 18 SP clusters were *unique* (observed only in any 1 condition) and (c) 32 SP clusters were *optional* (observed in 15–77% of conditions) (Fig. 4). This grouping was identified as following. Theoretically expected frequency of each SP cluster occurrence across 13 conditions should be 6.5 (50%). Observed occurrence frequency of 8 SP clusters across 13 conditions approached a theoretical value (Fig. 4, SP clusters that occurred in 6 and 7 conditions). Observed occurrence frequency for the rest of SP clusters was either higher or lower than theoretical one. Identification of unique SP clusters’ group is obvious: SP cluster should be observed only in any one condition. For the group of the optional SP clusters we choose equal number SP clusters (±12) which has higher and lower observed frequency of occurrence than theoretically expected frequency (considering that at the lower end it should reach the unique group) (Fig. 4). The remaining SP clusters at the upper end form the universal group that included only SP clusters that were observed in more than 85% of conditions (Fig. 4). If our grouping is correct then observed distribution of the SP clusters into these categories should be different from a chance distribution. A Chi-square test revealed that distribution of the SP clusters into these categories represent statistically significant deviation from a chance distribution (*p*<.001).

Analysis of the morphology of SPs separately for unique, optional and universal SP clusters revealed that SPs of different morphology dominated in each of these groups (Table 1). Thus, SPs with 2 and 3 main frequency peaks prevailed in unique SP clusters (50% for 2 peaks and about 40% for 3 peaks). SPs with 1 and 2 main frequency peaks dominated in optional SP clusters (31% for 1 peak and 47% for 2 peaks). Finally, SPs with 1 main frequency peak dominated in universal SP clusters (63%) (Table 1).

**Table 1:** The number of clusters of spectral patterns with different morphology among (a) unique SP clusters (observed in any 1 (8%) condition), (b) optional SP clusters (observed in 15–77% of conditions) and (c) universal SP clusters (observed in more than 11 (≥85%) conditions).

	unique SP clusters		optional SP clusters		universal SP clusters	
SP morphology	abs	%	abs	%	abs	%
one dominant peak	2	11	10	31	5	62.5
two dominant peaks	9	50	15	47	2	25
three dominant peaks	7	39	5	16	–	–
four dominant peaks	–	–	2	6	–	–
Polyrhythmic	–	–	–	–	1	12.5
Total	18 = 100%		32 = 100%		8 = 100%	

#### 2.3. SP cluster types

**Figure 6:**
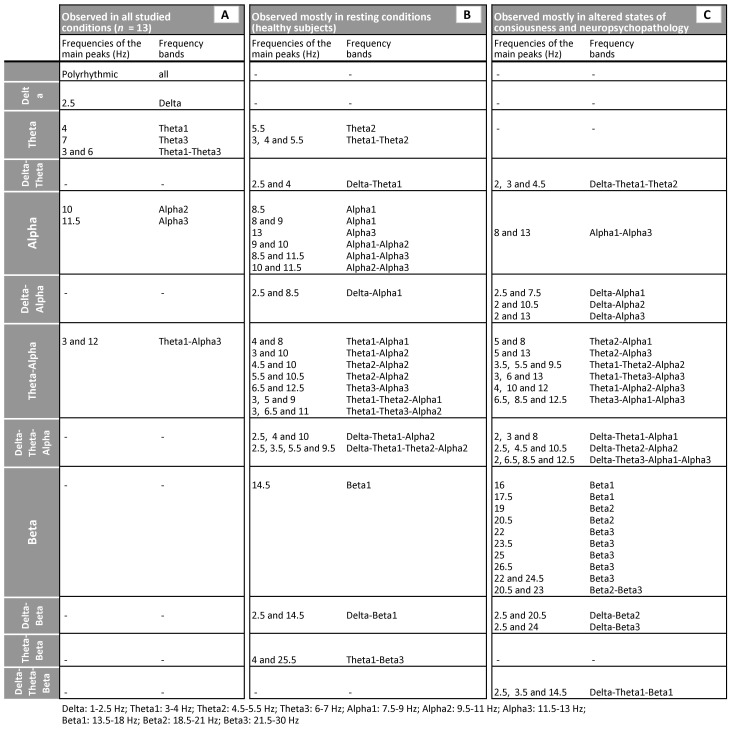
Clusters of spectral patterns. List A: clusters of spectral patterns which are universal for all studied conditions. List B: clusters of spectral patterns which are observed mostly in resting conditions. List C: clusters of spectral patterns which are observed mostly in altered states of consciousness and neuropsychopathology. “–” = none of the SP clusters in a given frequency range was observed.

To estimate which EEG oscillations (within a broad frequency range of 1–30 Hz) occurred more or less frequently across groups of conditions, we examined the frequency of each SP cluster type occurrence (which characterise EEG oscillations and/or their mixture) (Fig. 6).

Analysis revealed that all 13 conditions were characterised by the same five EEG oscillations (delta, theta_1_, theta_3_, alpha_2_, and alpha_3_) alone or combined with each other as well as *polyrhythmic* activity (measured as 8 universal SP clusters) in different EEG segments, thus exhibiting “mosaic” dynamics (Fig. 6, A). Polyrhythmic activity (presented by a pattern where peaks occupy the majority of the frequencies within the studied range) indicates a mixture of activity of small neuronal subpopulations each with its own mode [97]. In addition to these five universal EEG oscillations and polyrhythmic activity, the group of resting conditions in healthy subjects was characterised by two additional EEG oscillations: theta_2_ and alpha_1_ (Fig. 6, B). The most diverse combinations of EEG oscillations were in alpha and theta-alpha frequency bands during resting condition in healthy subjects.

Group of altered states of consciousness and neuropsychopathology was characterised by additional three (beta_1_, beta_2_ and beta_3_) EEG oscillations besides the five universal EEG oscillations (Fig. 6, C). EEG oscillations alone or in combination from theta-alpha and beta frequency bands were present the most in this group of conditions.

## Discussion

### 1. Parameters of Dynamic Repertoire of EEG Oscillatory States and the Frequency of Each Oscillatory State Occurrence during Resting Condition with Closed Eyes in Healthy Subjects

We start our analysis by considering the resting condition with eyes closed in healthy subjects. The resting brain state with eyes closed in healthy subjects constitutes a reference baseline, relative to which many cognitive and physiological brain states are usually considered [98], [99].

Analysis of the repertoires of SP clusters for resting conditions with eyes closed from four experimental studies revealed that multichannel EEG total variability during multiple realisations of the resting condition is limited by 30 types of local oscillatory states in healthy subjects (Fig. 3). Each realisation of the resting condition was characterised by smaller repertoire of local EEG oscillatory states: from 9 to 26. Recall that in this study a repertoire of EEG oscillatory states includes state types found in all EEG channels during a given condition. The size of the repertoires of EEG oscillatory states for each EEG channel is ranged in more narrow limits: from 5 to 16 types for different conditions [45], [56], [58], [61], [62], [66], [65]. This is consistent with the earlier findings obtained by authors who used spectral analysis for characterisation of EEG states within separate EEG channels [40], [51]. This can be interpreted as the brain “operates” via a fixed number of oscillatory states which are produced by different but constrained configurations of firing neurons (for the relations between EEG SP and the actual state of the neurons in the underlying network, see appendix in [46]). This is in line with previous studies that have demonstrated that an individual (local) EEG signal is described by a stable and restricted set of SP types [1], [31], [40], [45], [46], [51]. Different authors have reported different number of distant EEG segment types: from 5 to 35 [1], [40], [41], [45], [46], [51]. The number of types of EEG segments depends on (a) the number of EEG channels analysed, (b) the mean age of subjects’ sample used, (c) the number of conditions examined and (d) classification criteria used: spectral power, frequency of the main peaks in the power spectrum, overall shape of the power spectrum and so on. It is natural that each of these criteria capturing somewhat different aspects of real functional “modes” of cortical activity would result in different number of distant EEG segment types.

The significance of these EEG oscillatory states for resting condition with eyes closed in healthy subjects was not equal: the frequency of each of these oscillatory states occurrence for each studied manifestation of the resting condition varied. Thus, six (20%) SP clusters from this common repertoire were *universal* (each appeared for all resting conditions), suggesting that 6 oscillatory states with delta-, theta_1_-, theta_2_-, alpha_2_- and alpha_3_- oscillations alone or in combination, as well as polyrhythmic activity are the necessary elements of neurodynamics during each studied manifestation of resting condition with eyes closed (Fig. 3). Nineteen (63%) SP clusters from common repertoire were *optional* (each appeared for 50–75% of resting conditions), whereas five (17%) SP clusters were *unique* – each appeared for only one manifestation of the resting condition with eyes closed (Fig. 3). Therefore we could conclude that individual peculiarity of an EEG for a particular manifestation of resting condition with closed eyes in healthy subjects is determined by varying proportions of universal, optional and unique oscillatory states.

The size of these dynamic repertoires of EEG oscillatory states during resting condition with eyes closed in healthy subjects was age dependent: it decreased as subjects’ age increased (Fig. 3). This suggests that EEG phenomenological variability during rest decreased with age. Similar relationship was found by Garrett et al. [100]. On the one hand, observed correlation may reflect age-related changes in the brain. Indeed, after adolescence, a steady decrease in brain volume occurs, which appears to become more severe with age [101]; [102]. Pakkenberg and Gundersen [103] reported a loss of about 10% of neurons in the neocortex of both males and females between age 20 and 90. Thalamic volume also decreases with age, either due to loss or shrinkage of neurons [104]; [105]. Additionally, gains for excitatory and inhibitory cortical interactions decrease until about age 40 [106], whereas the number of GABAergic synapses increases throughout this period of life [107]. On the other hand, age-related decrease in variability of EEG may reflect an increased dominance of refined and stereotyped psychological patterns in a subject’s behaviour with age.

Taken together, the presented findings suggest that the resting condition with eyes closed is very much an active state [31], where local EEG oscillatory states emerge, persist for some time and then disappear to be replaced by other oscillatory states within each EEG channel. The diversity of these states is limited and functionally heterogeneous. This suggests that there is a limit in the number of locally accessible oscillatory states available to the cortex and many different ways in which the microstates (indexed by the types of SP clusters) can rearrange themselves in each EEG channel and between the channels, and still produce the same macrostate – a resting condition. Thus, ongoing brain activity reflects the poly-operational structure of resting brain activity (for discussion see [31]) and confirms that the cerebral cortex is continuously active in wakefulness. This supposition is in line with the works of Thatcher and John [108], Herscovitch [109], Arieli et al. [110], Tsodyks et al. [111], Raichle et al. [112], Raichle and Snyder [99] and others who demonstrated a highly organized intrinsic functional activity during a resting state i.e., activity which is not directly related to identifiable sensory or motor events. Recent support for this view came from Fingelkurts and Fingelkurts study [113] which presented evidence that the resting state with eyes closed is characterised by dynamic functioning of relatively large, but relatively short-lived neuronal assemblies and certain level of functional connectivity among them.

### 2. Parameters of Dynamic Repertoires of EEG Oscillatory States and the Frequency of Each Oscillatory State Occurrence Across Diverse Healthy and Pathological Conditions

The common repertoire of EEG oscillatory states for all studied manifestations of resting condition with eyes closed (across different age groups) served as a reference state against which all other conditions were contrasted. This reference condition was normalized for the age factor. Thus, it allows us to compare different groups in which subjects with different ages (from 19 to 60 years) participated.

#### 2.1. Size of the repertoires of EEG oscillatory states (indexed by SP clusters)

Size of the repertoire of EEG oscillatory states (expressed as a multitude of SP clusters from the same and different EEG channels) represents the range of the probable local states available to the cortex. All phenomenological manifestations of EEG across 13 diverse conditions which cover healthy rest, cognitive tasks, altered states of consciousness and pathological states were described in total by a multitude of 58 types of oscillatory states (measured as the number of SP cluster’s types, Fig. 4). EEG in each and every condition was characterised by a limited repertoire of oscillatory states which ranged from 13 (rest during depression) to 30 (healthy rest) SP cluster types (Fig. 4). The largest decrease in the repertoires size of oscillatory states was observed during neuropsychopathologic conditions, a moderate decrease was during cognitive loading and smallest – during altered states of consciousness conditions (Fig. 5). The following is a separate consideration of each of the group conditions.

A progressive increase in cognitive loading [114] resulted in considerable narrowing of correspondent repertoires reaching a minimum during information memorisation (M) and retention (K) (Fig. 5, A). This is not surprising: closed eyes resting state is a state of relaxation and non-focused attention where cortical processes and associated internal mental activity are not determined by external stimuli but are driven by free floating associations, mental imagery, planning, etc, and random shifts from one mental object or theme to another are present. Such internal mental activity (top-down processing) was probably reflected in the wide repertoire of EEG oscillatory states. Opening the eyes resulting in nonspecific activation caused by basic sensory input was reflected by a decreased number of accessible local oscillatory states available to the cortex. This can be explained that only a subset of resting oscillatory states is needed to aid the processing of visual information on the one hand [115] and provide general task demands and attentional processes on the other hand [116], [117]. The following “waiting” (W) condition which is characterized by mobilization of resources with alertness, arousal and readiness to process information was associated with further reduction in the repertoire size of oscillatory states. Memorizing (M) and retention (K) conditions being conditions with highest focused attention were characterized by the smallest number of accessible oscillatory states available to the cortex. Whereas the retrieval (R) condition (where several recognition and comparison operations are involved) was characterized by a widening of the size of repertoire of oscillatory states when compared to previous conditions.

Altered states of consciousness (natural sleep, hypnosis and benzodiazepine-induced sedation) were also characterised by a reduction in repertoire size of oscillatory states (Fig. 5, B), thus suggesting less diverse and less variable EEG (both temporally and spatially) when compared with the healthy resting condition with closed eyes. This can be interpreted as a decrease in the number of information processing modes, thus suggesting a reduction in brain information processing [118] with a larger reduction for artificially-induced sedation conditions.

Finally, all examined neuropsychopathologic resting conditions demonstrated considerably smaller repertoires of EEG oscillatory states when compared with healthy resting condition (Fig. 5, C). Among neuropsychopathologic resting conditions the smallest repertoire of oscillatory states was observed in the depressive (D) condition and the largest in the “opioid withdrawal” (OW) condition. It can be suggested that the severity of neuropsychopathology is associated with the degree to which repertoire size of EEG oscillatory states decreases. However, the number of predominantly impaired brain systems for each condition [96] correlated negatively with the repertoire size of oscillatory states (Fig. 5, C). Therefore, more studies are needed to clarify the relationship between repertoire size of oscillatory states, number of the predominantly impaired brain systems and the degree of neuropsychopathology. Considerable decrease in EEG variability for neuropsychopathologic conditions corroborates with a well known fact that a loss of variability in the majority of physiological measures is associated with an increased pathology [119]–[121]. It seems that none of the studied neuropsychopathologic condition could reach a proper (for the healthy brain) resting state. As a result, such a system is less able to cope with the demands of a constantly changing environment. Decreased repertoires size of EEG oscillatory states during neuropsychopathologic conditions may reflect alteration in neuronal assemblies functioning. This suggestion is supported by an earlier study which demonstrated that the size, stability and functional life-span of neuronal assemblies were increased and reached “true” pathological values in depression, opioid abuse and abstinence pathological conditions when compared with the healthy reference functional state [113]. By “*true*” pathological values we mean the values which are outside the boundaries of variability of healthy resting state with closed eyes. These enlarged, stable and long-lived neuronal assemblies in the mentioned neuropsychopathologic conditions can be interpreted partially in terms of persistent and recurrent presence of the same train of limited thoughts: negative thoughts in depression that maintain depressive affect and cognition [122] and drug-related thoughts that lead addicts to drug-seeking and drug-taking behavior [123] in opioid abuse and abstinence. One may speculate that decreased repertoires size of EEG oscillatory states during neuropsychopathologic conditions reflect a situation where both neural and thought dynamics getting “stuck” in so-called attractor states.

#### 2.2. Uniqueness and universality of EEG oscillatory states (indexed by SP clusters)

Analysis of the frequency of each oscillatory state occurrence across 13 diverse conditions covering healthy and pathological states revealed that (a) eight (14%) EEG oscillatory states were *universal* (observed in more than eleven (≥84%) conditions), (b) eighteen (31%) EEG oscillatory states were *unique* (observed once in any one condition) and (c) thirty-two (55%) EEG oscillatory states were *optional* (observed in 15–77% of conditions) (Fig. 3). The number of universal EEG oscillatory states observed in this study (8 types) is within the range observed earlier: from 6 [40] to 11 [45] types of universal local EEG oscillatory states.

Perhaps universal EEG oscillatory states reflect the general properties and necessary oscillatory elements of neurodynamics that are independent of the brain’s functional state, cognitive task, health or neuropsychopathology. It is very likely that universal EEG oscillatory states are determined genetically. Indeed, the heritability of these EEG oscillations is estimated to be between 80% and 90% [124]; for a review and meta-analysis see [125]) and these EEG oscillations share a common genetic source ([126]; see also [127]). This common genetic source for EEG oscillations may reside in the common influences on the cerebral rhythm generators or in genes directly involved in the bioelectric basis of the EEG signal itself [126] by influencing the number of pyramidal cells, amount of dendritic connections and their orientation with respect to the scalp [128]. Additionally, it was reported that the ratio of polyrhythmic activity in the EEG is also strongly influenced by genetic factors [129].

Unique EEG oscillatory states are most likely related to a few limited functions which are necessary for a given condition. Therefore, the individual peculiarity of an EEG within any of its manifestations is determined by varying proportions of universal, optional and unique oscillatory states [40]. Such structural-functional organisation of brain activity, where the proportion of universal, optional and unique EEG oscillatory states is dynamic, meets the complex computational and communicational demands of the brain by flexibly modifying this proportion in a wide variety of ways.

Considering extensive data on how SP morphology depends on neurophysiologic parameters and nonlinear measures ([29], [97], [130]–[135]; to mention just a few), our data on morphology of SP clusters can be interpreted in terms of states of the underlying neuronal assemblies. Analysis of the morphology of SPs clusters separately for unique, optional and universal SP clusters (Table 1) revealed that unique EEG oscillatory states were generated mostly by two (in 50%) or three (in 40%) neuronal ensembles with resonant ordered behaviour within each individual ensemble [97], [130], [133]. Optional EEG oscillatory states were produced mostly by one (in 31%) and two (in 47%) neuronal ensembles, whereas universal EEG oscillatory states were generated mostly (in 63%) by a single neuronal ensemble [133], [134], which is is characterised by resonant ordered behaviour with low entropy [97], [130], [132] in a short-term temporal scale.

#### 2.3. Type of EEG oscillatory states (indexed by SP cluster types)

Nowadays a large body of knowledge has accumulated on functional significance of EEG oscillations (for the review see [47]; see also ref. list of this article). Considering that different EEG oscillations reflect functionally different components of information processing acting on various temporal scales [136], [137] it is possible to map EEG oscillations onto mental and/or behaviour states [138].

Analysis of EEG oscillations which contributed to EEG oscillatory states within groups of conditions revealed that universal EEG oscillatory states were characterised by delta, theta_1_, theta_3_, alpha_2_, and alpha_3_ EEG oscillations alone or in combination and by polyrhythmic activity in different EEG segments, thus exhibiting a “mosaic” dynamics (Fig. 6, A). All these EEG oscillations have a number of universal functions, which may explain participation of these oscillations in the universal EEG oscillatory states: (a) delta oscillations are associated with states oriented to the acquisition of biologically important goals such as physical maintenance, survival, dominance and mating [139], [140], (b) theta oscillations are expected to be associated with emotional regulation [141]–[143] and episodic memory demands [115], [144], [145], (c) alpha oscillations are involved in the organization of conscious interactions with the environment [140], [146] and associated with spontaneous self-referential thoughts [147] and (d) polyrhythmic activity is necessary to maintain a high level of activity in neuronal networks for sustained periods of time [148].

In addition to oscillations of the universal EEG oscillatory states, the group of resting conditions in healthy subjects was characterised by two additional EEG oscillations: theta_2_ and alpha_1_ (Fig. 6, B). The most diverse combinations of EEG oscillations were in the alpha and theta-alpha frequency bands for resting conditions in healthy subjects. A number of specific functions of all these EEG oscillations which may be useful for resting conditions, where cortical processes and associated internal mental activity are not determined by external stimuli, but driven by free floating associations, mental imagery, planning, etc [117], [149], [150], are the following: (a) delta oscillations are related to states which require attention to internal processing [151], (b) theta oscillations reflect control processes that may operate under top-down control or in a default-like mode [152] and may serve as a response controlling function [137], (c) alpha_1_ oscillations are related to general non-focused attentional demands [115], whereas alpha_2_ oscillations may facilitate association mechanisms in the brain [153] and participate in instantaneous recognition of environmental patterns by means of matching them with categorized knowledge stored in semantic memory [139], [152], (d) polyrhythmic activity representing stochastic resonances are an important mechanism by which very small signals can be amplified and emerge from the random noise of physiological oscillations [154].

The group of altered states of consciousness and neuropsychopathologic conditions was characterised by three more EEG oscillations (beta_1_, beta_2_ and beta_3_) in addition to oscillations of the universal EEG oscillatory states (Fig. 6, C). EEG oscillations alone or in combination from theta-alpha and beta frequency bands were the most prominent in this group of conditions. The following specific functions of all observed EEG oscillations may explain their dominance in EEG oscillatory states during altered states of consciousness and neuropsychopathology: (a) delta oscillations have been related to increased inhibition such as sleep or states requiring attention to internal processing in healthy subjects [151] or to opioid addiction in opioid-abusing individuals [155], [156], (b) theta oscillations are associated with a hypnagogic state in which individuals are drowsy and have a marked decrease in awareness of their environment [157], [158] or are associated with impulsive behaviour [139], [140], (c) alpha_2_ and alpha_3_ oscillations suggest an increase in alertness and can be interpreted as reflecting increased excitation of neuronal ensembles on the one hand and higher readiness to respond to stimuli (anxiety) on the other hand [159], [160], (d) beta oscillations are considered as an index of a higher level of cortical activation and irritation [96], [159]–[161] and related to dysfunction in GABA_A_ receptor genes which underlies the imbalance between excitation–inhibition (hyperexcitability) and is involved in the predisposition to develop pathological dependencies and other disinhibition disorders [161], [162], (e) polyrhythmic activity at elevated levels is a sign of pathological processes [48], [163]; the fact that the frequency spectrum becomes increasingly peaked as the system approaches a change of state [164], [165] suggests that the amount of polyrhythmic activity would increase while approaching an epileptic seizure and that a higher percentage of polyrhythmic activity is likely to increase the probability of a seizure [58].

Notice that there was no single SP type which would have a dominant narrow peak in a beta frequency band in resting conditions and studied cognitive tasks in healthy subjects (Fig. 6, A, B). This does not mean that these conditions do not have beta oscillations. However it means that during each observation (2 sec) beta oscillations are not dominant in relation to delta, theta and alpha, suggesting that independent beta rhythm is a less probable oscillation during these conditions. This is consistent with the work of Simon [166] who founds beta frequencies in only 22% of normal adults. Well known beta activity visible in averaged power spectrum is most likely a result of the contribution of the averaging of the polyrhythmic SPs which have power peaks at beta frequency band along with other peaks at other frequency bands. Additionally, beta activity in resting conditions and cognitive tasks in healthy subjects is characterised often by non-dominant very broadband peak (spectral power peaks at other frequency bands are dominant) rather than a well-defined narrow peak. In contrast, EEG from neuropathological conditions and altered states of consciousness is characterized by diverse SP types with dominant well-defined narrow peaks in beta band (Fig. 6, C).

Taking the aforementioned results and literature data together it is possible to suggest that each EEG oscillation is related to multiple functions (universal and specific) and a given function is often manifested by means of multiple EEG oscillations [167], [168] which are organised in a particular proportion of universal, optional and unique EEG oscillatory states. Thus, EEG oscillations from the same frequency band may express different functions depending on conditions they involved in. This seems biologically plausible: EEG oscillatory functional diversity creates a rich repertoire of brain activity, which can meet the complex computational and communicational demands of the brain. By preventing neural dynamics from getting “stuck” in so-called attractor states, neural diversity may facilitate quick responses to environmental demands in a wide variety of ways, and with less effort than a system where all states are identical.

Percent ratio of each oscillatory type of EEG states for each EEG channel during each studied condition together with functional interpretation is beyond the scope of this study and can be found in correspondent studies (see references provided for each used study above).

Before coming to the final conclusions, methodological question regarding the influence of reference electrode on SP shape should be raised. One may assume that some of the obtained results may be affected by the fact that in some studies linked-ears were used as EEG reference and in others nose was used as EEG reference. However it is unlikely since analysis of local signals for EEG (reference-dependent technique) and MEG (reference-free technique) registered simultaneously revealed the same classes of SP types, very similar percent ratio of these classes and very similar temporal stabilization of SPs between EEG and MEG [61]. This is due to fact that reference choice influences magnitude and distribution of power spectra but not the shape of SP [169], [170], which is essential for our analysis.

Limitation of the Study-8 that presents hypnosis condition should be mentioned. In this study only one virtuoso subject (very highly susceptible individual) was participated (even though many EEGs were recorded), therefore results from this study should be considered with caution. However there are several advantages of this single case study: (a) The typical way in the field to operationalize hypnosis is to use scales which measure so-called hypnotic susceptibility. The responses measured or assessed by most of these scales concern overt behaviour rather than subjective experiences. In such a way the search for neurophysiological correlates is performed in the heterogeneous group of subjects, which have an identical score obtained on a given behavioural scale, but different underlying neuropsychophysiological processes. Therefore a single case study is free from such group averaging; (b) The usage of virtuoso permitted us to study hypnosis state in its pure form free from the influences of standard experimental induction procedures and suggestions in particular contents of consciousness (See Details in [66]). Therefore the ‘hypnotic’ phenomenon in the Study-8 is manifested in it clearest form and is not easily confused with any other phenomena, such as simple compliance and faking, or with relaxation suggestions and hypnotic hallucinations, or with “virtual” phenomena resulted from group averaging. Additionally, oscillatory content of this virtuoso’s EEG was the same as reported for other virtuosos. Taking together, the abovementioned advantages and typicality of spectral description of EEG for virtuosos justify presentation of these data.

## Conclusions

This is the first EEG study that covers so diverse and broad range of conditions using the same methodological and conceptual framework in order to quantify the dynamic repertoires and oscillatory types of local EEG states. This methodological approach enabled us to reveal peculiarities and generalities of EEG oscillatory states across a multitude of different conditions and tasks which cannot be seen within any one study:

- The observed results demonstrate that brain activity (either within individual EEG channel or in all EEG channels taken together) consists of a *limited* repertoire of EEG oscillatory states in any of the 13 examined conditions; and this repertoire is ranges from 13 (rest during depression) to 30 (healthy rest) SP cluster types. This suggests that there is a limit in the number of accessible oscillatory microstates available to the cortex (either locally or globally) and many different ways in which these microstates can rearrange themselves and still produce the same macrostate. Even resting condition with eyes closed is very much an active state with the *poly-operational* structure, where EEG oscillatory states emerge, persist for some time and then disappear to be replaced by other oscillatory states within each EEG channel.
- The repertoire’s size of oscillatory states was associated with cognitive or vigilance states changes or neuropsychopathologic conditions. Thus, the largest size of the repertoires of oscillatory states was observed for altered states of consciousness, the medium size was during cognitive loading states and the smallest size was found for neuropsychopathologic conditions. At the same time, all these conditions were characterized by smaller size of the repertoires of oscillatory states compared to the baseline resting condition with closed eyes.
- Not all EEG oscillatory states occur with the same frequency – some of them seem to be “preferred” within and across conditions. The existence of *universal*, *optional* and *unique* EEG oscillatory states across 13 diverse conditions was observed. It is very likely that universal EEG oscillatory states are associated with functions which are invariant and necessary for any condition (acquisition of biologically important goals such as physical maintenance, survival, dominance, mating, emotional regulation and organization of conscious interactions with the environment) and therefore they should be determined genetically. On the contrary, unique EEG oscillatory states are likely to be related to more specific functions which manifest themselves only under certain conditions. Therefore, individual peculiarity of an EEG within any of its manifestations is determined by varying proportions of universal, optional and unique oscillatory states.
- The dynamic repertoires and oscillatory types of local EEG states identified in the present study possess most likely distinct trait-like qualities. Using the same methodological approach it was demonstrated earlier [46] that the size of repertoires of EEG states, oscillatory types of EEG states and there percent ratio (i) were highly stable across all one-min EEGs for each subject during resting conditions and during memory task and (ii) demonstrated very high test–retest reliability for resting conditions and the memory task. Moreover, the reliability values demonstrated regular changes in accordance with the changes of functional brain state and stages of the memory task, thus exhibiting functional relevance [46]. These findings might be the manifestation of intra-individual stability of neurodynamics and underlying regulatory mechanisms. Additionally, the size of repertoires of EEG states, oscillatory types of EEG states and there percent ratio were typical for each of the examined conditions (for similar view see [40]).
- Joint analysis of the results obtained in this study and previously published data suggested that unique EEG oscillatory states were generated by mostly two or three neuronal ensembles with resonant ordered behaviour within each individual ensemble. Optional EEG oscillatory states were produced by mostly one or two neuronal ensembles, whereas universal EEG oscillatory states were generated by mostly a single neuronal ensemble which was characterised by resonant ordered behaviour with low entropy.
- Additionally it was proposed that EEG oscillations which constituted EEG oscillatory states are characteristic for different groups of conditions according to functional significance of these EEG oscillations.

In future research it will be important to establish the repertoires of mental and cognitive operations which accompany the types of observed EEG oscillatory states.

## Supporting Information

Appendix S1 **Relation of local short-term spectral patterns (SPs) and local EEG oscillatory states.** Discussion of the relation of local short-term SPs and local EEG oscillatory states and the brief review of the empirical data on the contribution of volume conduction to the local EEG is presented.
(DOCX)
