# Supplementary material for: EEG Oscillatory States: Universality, Uniqueness and Specificity across Healthy-Normal, Altered and Pathological Brain Conditions
Source: PLoS One. 2014 Feb 5;9(2):e87507. doi: 10.1371/journal.pone.0087507 (PMC3914824; doi:10.1371/journal.pone.0087507)
Supplement: Appendix S1 — Relation of local short-term spectral patterns (SPs) and local EEG oscillatory states. Discussion of the relation of local short-term SPs and local EEG oscillatory states and the brief review of the empirical data on the contribution of volume conduction to the local EEG is presented. (DOCX) [file pone.0087507.s001.docx]

**Appendix S1**

***Relation of local short-term SPs and local EEG oscillatory states***

The usage of local short-term SPs for characterisation of local EEG oscillatory states is justified due to the following reasoning:

1. A single EEG spectrum reflects the coordinated activity of tens and hundreds of thousands of neurons at a particular point in time [1]. However, there is no simple (one-to-one) relation between a power spectrum computed from short epochs of ongoing EEG and the actual state of the neurons in the underlying network: many different configurations of firing neurons can give rise to a particular short-term SP (many-to-one relation). At the same time, *the same configuration of firing neurons cannot give rise to two (or more) different short-term spectra*. Thus, two different short-term power spectra most likely are originated from two different configurations of firing neurons [2]. Consequently, short-term SP characterises/reflects a particular class of neurons’ activities, where each of the activities has something common with the others within the class (one-class–to–one relation). Moreover, two classes of neurons’ activity do not overlap (otherwise the same configuration of firing neurons could give rise to two or more different short-term spectra). Thus, a given type of short-term SP may be considered as a single event (which reflects a particular class of neurons’ activity) in EEG phenomenology from viewpoint of its spectral characteristics [3]. In this context it can be suggested that the SPs within each class are generated by similar neurodynamics class as well as driving force [2]. SPs from different classes, however, are expected to have different driving forces and therefore generated by different neurodynamics classes. Thus, each perceptual, cognitive, or mental operation is thought to constitute a single distinguishable neurophysiological state with a distinct and reliable SP type [4-7]. Therefore, the abrupt transition from one SP type to another reflects a change in the transient neuronal assembly state or changes in the activity of the two or more neuronal assemblies [8]. In this case, the frequency of appearance of each SP type reflects the probability for the occurrence of particular neuronal dynamics class, which constitute a dynamic repertoire of brain activity under a particular functional state or condition.
2. It is often claimed that volume conduction is the main obstacle in interpreting local EEG data: each EEG electrode registers activity from many sources – in other words, locally registered EEG activity is a result from a mixture of volume conduction effect and genuine local source activity. However, *it is often ignored that only some sources contribute to local EEG considerably and others insignificantly*. What is the contribution of volume conduction effect in this context?

Firstly, volume conduction effect is distance dependent: the larger the distance of the recording electrode from the current source, the less informative the measured potential becomes about the events occurring at the location(s) of the source(s) [9]. Secondly, superficial sources contribute a strong potential that is restricted in extent to nearby electrodes on the scalp, and are thus the most likely sources to be accurately localized with scalp recordings [10]. It has been estimated that one electrode integrates cortical input under a scalp surface of the order of 6 cm^2^ [11-14]. Therefore, *at distances greater than 4 cm volume conduction effect is predicted to be very small* [14].

Such prediction is supported by experimental findings which suggested the existence of statistical heterogeneity (anisotropy) of electromagnetic field in regard to the processes in local LFP [15] and local EEGs [16-20]. It was demonstrated that such *electromagnetic heterogeneity relates to large-scale morpho-functional organization of the cortex*:

1. Covariance between neighboring electrodes across cortex functional boundaries (e.g., parietal to temporal areas) is much smaller than covariance within functional regions (e.g., left parietal to midline parietal area), indicating that multiple distinct functional areas are assessed by topographic EEG [21; 22]. This morpho-functional heterogeneity of EEG was also confirmed in independent study in which the spatial heterogeneity of scalp-recorded EEG synchronicity was measured along longitudinal (the anterior-to-posterior and posterior-to-anterior directions) and transversal (right-to-left anterior and right-to-left posterior directions) electrode arrays with scalp electrodes equally spaced in all these arrays [17]. Data from actual EEG was compared with so-called “surrogate” EEG in which a mixing of actual local EEG recordings was done in such a way that each local recording was registered in a different time so that the natural time relations between all local EEG recordings in such EEG were completely destroyed, however, the number and the sequence of segments within each local recording remained the same as in the actual EEG. For longitudinal electrode arrays, despite the fact that all testing pairs of EEG electrodes had the same interelectrode distance, synchronicity index exhibited the notable topological landscape: it significantly decreased in locations of EEG electrode pairs on the head which overlay cortex functional boundaries [17]. This data clearly indicate that at the boundaries of well-outlined functional cortical areas the temporal consistency of segmental architectonics of electrical field becomes weak. Additionally (i) the relationship between synchronicity index and interelectrode distance was not monotonous for both longitudinal electrode arrays: step-wise dependency was observed and (ii) forward (posterior-to-anterior) and backward (anterior-to-posterior) dependences of synchronicity index from the interelectrode distance were significantly differing between each other [16, 17]. These results suggest that volume conduction role here is insignificant. For transversal electrode arrays it was demonstrated that (i) anterior and posterior cortex areas had opposite tendencies in the dynamics of synchronicity index (notice that anterior and posterior cortex areas have different morpho-functional organisation) and (ii) maximal synchronicity index values in the posterior cortical areas were obtained for homological lateral EEG locations (which have similar morpho-functional organisation) in spite of the largest interelectrode distance in the electrode array [16, 17];
2. The probabilities of firing of neurons observed singly and in small groups simultaneously are in close statistical relationship to the EEG recorded in the near vicinity [23, 24]. Therefore local EEG can provide an experimental basis for estimating the local mean field of contributory neurons;
3. The accuracy of topographic EEG mapping for determining local (immediately under the recording electrode) brain activity was demonstrated [184, 185]: there are statistically significant linear relationships between local EEG power and cerebral perfusion underlying the electrode in the majority of frequency bands [25, 26]. These findings are in line with earlier study of Inouye et al. [27], where the authors demonstrated that endogenous EEG activity originated from underlying cortex area contributes the most to the spectral power measured from the given EEG electrode. Whereas exogenous EEG activities originated from the other cortical areas contribute to spectral power of the same EEG electrode insignificantly. Thus, together described works suggest that topographic EEG mapping can accurately reflect local brain function and that it is comparable to other topographic methods;
4. Each local EEG or small group of local EEGs are characterised by a relatively specific set of SP types [28] presumably due to their different degree of involvement in the condition or the task;
5. The same type of SP is usually observed simultaneously within the same observation in two functionally homologous EEG channels: for example O1-O2 [29], thus suggesting functional topology of SP types rather than volume conduction effect;
6. Cortex areas separated by distances exceeding the diameters of ‘wave packets’ have differing wave forms and therefore different SP types [30, 31]. The coordinated activity manifests a ‘wave packet’ that requires synchronization of a shared carrier wave of the outputs of a large number of neurons over the area [30, 31];
7. Topographically specific modulation of local EEG rhythms by direct cortical stimulation via TMS in TMS–EEG studies have been demonstrated [32-35]. Such TMS-induced entrainment of local brain oscillations due to direct interaction with the underlying local generator revealed causal relations between local EEG oscillations and underlying local generator;
8. Compared to the EEG, the MEG is very little affected by the type and location of tissue surrounding the generator, and especially that of tissue lying between the generator and the sensor [36]. Additionally, the MEG is much more directly related to the intracranial currents and is therefore less sensitive to the details of the skull conductivity [37]. All these minimize volume conduction effect in MEG [38]. Analysis of local signals for EEG and MEG registered simultaneously revealed the same classes of SP types and very similar percent ratio of these classes and very similar temporary stabilization of SPs between EEG and MEG [39]. These results suggest that volume conduction effect on SP analysis based on power spectra shape is insignificant at least for 64-channel EEG.

There could be several reasons for these experimental results:

1. as the spatial resolution of EEG has been estimated to be approximately 2 cm [40] to 5 cm [14, 41] with an electrode spacing of approximately 7 cm as in the 10/20 System (used in the majority of EEG studies) volume conduction effect becomes less likely;
2. the spatial damping is very high, and thus global resonant modes play no significant part in the generation of wave activity [42; 43];
3. volume conduction does not spread all forms of activity [15];
4. spread of activity in the cortex is not uniform in all directions as it follows from measurements of tissue resistance [44], as well as from measurements of spread of activity parallel and perpendicular to the surface by means of microelectrode arrays [45, 46];
5. volume conduction in tissues overlying the cortex is found to affect the spectrum significantly only above about 25-30 Hz [47]. Note that most of the physiological rhythms and approximately 98% of spectral power lies below that limit [48], and has the highest signal-to-noise ratio;
6. the conductivity values of the tissue compartments of the head (white-matter, gray-matter, CSF, skull, and scalp) are not well-known, so that even an exact geometric model of the head is still only an approximate volume conduction model of the head [173]. Additionally, volume conduction models focus at physical and anatomical constrains but do not take into consideration physiological data, e.g. the activity (state) of cortex areas. However, macroscopic measurements in cortex revealed a frequency dependence of electrical parameters (the conductivity and permittivity) [49]. The extracellular medium is reactive in the sense that it reacts to the electric field by polarization effects [50]. Electric polarization influences the frequency-dependent electric properties of the tissue what allows the electrical parameters (the conductivity and permittivity) to depend on frequency, as demonstrated by macroscopic measurements [49, 51, 52]. Electric polarization is a prominent type of reaction of the extracellular medium to the electric field. In particular, the ionic charges accumulated over the surface of cells will migrate and polarize the cell under the action of the electric field [50]. This surface polarization phenomena can have important effects on the propagation of local field potentials [53]. Important, the electrical field produced by neural activity can influence it back: studies have found that (i) extremely weak fields (<0.5 mV/mm) are capable of significantly modulating activity at the network and single cell level, (ii) endogenous fields are involved in generating and maintaining neural oscillations and (iii) functional field effect interactions in the brain are shaped by the temporal dynamics of neural activity especially when relatively homogeneous populations of neurons are synchronously active (for the review see [54], see also [55]).

Considering that all activities (influences) from multiple primary sources are not just mixed, summed or averaged in a given cortex area, but are rather integrated within the current state (activity) of the given area [56, 57], the local EEG registered from that area is considered to represent a *functional source*, which is defined as the part or parts of the brain that contribute to the activity recorded at a single sensor [58, 59]. A functional source is an operational concept that does not have to coincide with a well-defined anatomical part of the brain, and is neutral with respect to the problems of localization of primary source and volume conduction [58, 59].

Considering the aforementioned findings one may suggest that local EEG short-term SPs are mainly determined by underlying neurodynamic (functional state) and type of SPs reflects mainly large-scale morpho-functional organization of the cortex rather than the effect of volume conduction at least for 10/20 System (used in the majority of EEG studies) which measures the main cortex lobes.

**References**

1. Dumermuth HG, Molinari L (1987) Spectral analysis of the EEG. Some fundamentals revisited and some open problems. Neuropsychobiol 17: 85-99.
2. Manuca R, Savit R (1996) Stationarity and nonstationarity in time series analysis. Physica D 99: 134-161.
3. Fingelkurts AlA, Fingelkurts AnA (2010a) Short-term EEG spectral pattern as a single event in EEG phenomenology. Open Neuroimag J 4: 130-156.
4. Bodunov MV (1985b) Correspondence of nonstationary properties of EEG and temporal characteristics of behaviour. Psihologicheskii Jurnal (Journal of Psychology) 6: 125-134 (In Russian).
5. Bodunov MV (1987) Peculiarities of temporal sequences of EEG stationary segments and speed characteristics of human behaviour. Psihologicheskii Jurnal (Journal of Psychology) 8: 48-56 (In Russian).
6. Gevins AS (1984) Analysis of the electromagnetic signals of the human brain: Milestones, obstacles, and goals. IEEE Trans Biomed Eng 31: 833-850.
7. Gevins AS (1986) Quantitative Human Neurophysiology. In: Hannay HJ, editor. Experimental techniques in human neuropsychology. New York: Oxford Press. pp 419-456.
8. Jansen BH, Cheng W-K (1988) Structural EEG analysis: an explorative study. Int J Biomed Comput 23: 221-237.
9. Buzsáki G, Anastassiou CA, Koch C (2012) The origin of extracellular fields and currents — EEG, ECoG, LFP and spikes. Nature Reviews, Neuroscience 13: 407-420.
10. Srinivasan R (1999) Methods to Improve the Spatial Resolution of EEG. Int J Bioelectomagn 1(1): 102-111.
11. Cooper R, Winter AL, Crow HJ, Walter WG (1965) Comparison of subcortical, cortical and scalp activity using chronically indwelling electrodes in man. Electroencephalogr Clin Neurophysiol 18: 217-228.
12. Gutman AM (1980) Biophysics of extracellular brain currents. Moscow: Nauka.
13. Epstein VM, Brickley GP (1985) Interelectrode distance and amplitude of the scalp EEG. Electroencephalogr Clin Neurophysiol 60: 287-292.
14. Nunez PL, Srinivasan R, Westdorp AF, Wijesinghe RS, Tucker DM, et al. (1997) EEG coherency: I: statistics, reference electrode, volume conduction, Laplacians, cortical imaging, and interpretation at multiple scales. Electroencephalogr Clin Neurophysiol 103(5): 499-515.
15. Bullock TH (1997) Signals and signs in the nervous system: The dynamic anatomy of electrical activity. Proc Natl Acad Sci U S A 94: 1-6.
16. Fingelkurts AnA, Fingelkurts AlA (2008) Brain-mind Operational Architectonics imaging: technical and methodological aspects. Open Neuroimag J 2: 73-93.
17. Kaplan AY, Fingelkurts AnA, Fingelkurts AlA, Borisov SV, Darkhovsky BS (2005) Nonstationary nature of the brain activity as revealed by EEG/MEG: methodological, practical and conceptual challenges. Signal Process 85: 2190–2212.
18. Freeman WJ, Holmes MD (2005) Metastability, instability, and state transition in neocortex. Neural Netw 18: 497–504.
19. Towle VL, Carder RK, Khorasani L, Lindber D (1999) Electro-corticographic coherence patterns, J Clin Neurophysiol 16: 528–547.
20. Freeman WJ, Holmes MD, West GA, Vanhatalo S (2006) Fine spatiotemporal structure of phase in human intracranial EEG. Clin Neurophysiol 117: 1228–1243.
21. Kooi KA (1971) Fundamentals of electroencephalography. New York: Harper & Row Publishers.
22. Bullock TH, McClune MC (1989) Lateral coherence of the electroencephalogram: a new measure of brain synchrony. Electroencephalogr Clin Neurophysiol 73: 479–498.
23. Freeman WJ (1975) Mass Action in the Nervous System. New York: Academic Press.
24. Eeckman FH, Freeman WJ (1990) Correlations between unit firing and EEG in the rat olfactory system. Brain Res 528: 238–244.
25. Cook IA, O’Hara R, Uijtdehaage SH, Mandelkern M, Leuchter AF (1998) Assessing the accuracy of topographic EEG mapping for determining local brain function. Electroencephalogr Clin Neurophysiol 107: 408–414.
26. Oishi N, Mima T, Ishii K, Bushara KO, Hiraoka T, et al. (2007) Neural correlates of regional EEG power change. Neuroimage 36(4): 1301-1312.
27. Inouye T, Shinosaki K, Yagasaki A, Shimizu A (1986) Spatial distribution of generators of alpha activity. Electroencephalogr Clin Neurophysiol 63: 353–360.
28. Fingelkurts AlA, Fingelkurts AnA, Kaplan AYa (2003b) The regularities of the discrete nature of multi-variability of EEG spectral patterns. Int J Psychophysiol 47(1): 23-41.
29. Fingelkurts AlA, Fingelkurts AnA (2010a) Short-term EEG spectral pattern as a single event in EEG phenomenology. Open Neuroimag J 4: 130-156.
30. Freeman WJ (2003b) The wave packet: an action potential for the 21st Century. J Integr Neurosci 2: 3-30.
31. Freeman WJ (2004) Origin, structure, and role of background EEG activity. Part 1. Analytic amplitude. Clin Neurophysiol 115: 2077-2088
32. Paus T, Sipila PK, Strafella AP (2001) Synchronization of neuronal activity in the human primary motor cortex by transcranial magnetic stimulation: an EEG study. J Neurophysiol 86: 1983–1990.
33. Fuggetta G, Fiaschi A, Manganotti P (2005) Modulation of cortical oscillatory activities induced by varying single-pulse transcranial magnetic stimulation intensity over the left primary motor area: a combined EEG and TMS study. Neuroimage 27: 896–908.
34. Van der Werf YD, Paus T (2006) The neural response to transcranial magnetic stimulation of the human motor cortex. I. Intracortical and cortico-cortical contributions. Exp Brain Res 175: 231–245.
35. Thut G, Veniero D, Romei V, Miniussi C, Schyns P, et al. (2011) Rhythmic TMS Causes Local Entrainment of Natural Oscillatory Signatures. Cur Biol 21: 1176–1185.
36. Hari R, Lounasmaa OV (1989) Recording and interpretation of cerebral magnetic fields. Science 244: 432-436.
37. Haueisen J, Ramon C, Eiselt M, Brauer H, Nowak H (1997) Influence of tissue resistivities on neuromagnetic fields and electric potentials studied with a finite element model of the head. IEEE Trans Biomed Eng 44: 727-735.
38. Schack B, Grieszbach UG, Nowak H, Krause W (1999) The sensitivity of instantaneous coherence for considering elementary comparison processing. Part II: similarities and differences between EEG and MEG coherences. International Journal of Psychophysiology 31: 241-259.
39. Fingelkurts AlA, Fingelkurts AnA, Kivisaari R, Pekkonen E, Ilmoniemi RJ, et al. (2004) The interplay of lorazepam-induced brain oscillations: Microstructural electromagnetic study. Clin Neurophysiol 115(3): 674-690.
40. Lachaux JP, Rodriguez E, Martinerie J, Varela FJ (1999) Measuring phase synchrony in brain signals. Hum Brain Mapp 8: 94–208.
41. Nunez PL (1995) Neocortical Dynamics and EEG Rhythms. New York: Oxford University Press.
42. Wright JJ (2000) Developing testable theories of brain dynamics: the global mode theory and experimental falsification. Behav Brain Sci 23: 414-415.
43. Robinson PA, Loxley PN, O'Connor SC, Rennie CJ (2001a) Modal analysis of corticothalamic dynamics, electroencephalographic spectra, and evoked potentials. Physl Rev E 63(4): 041909-1–041909-13.
44. Nicholson PW (1965) Specific impedance of cerebral white matter. Expl Neurol 13(4): 386–401.
45. Elul R (1962) Dipoles of spontaneous activity in the cerebral cortex. Exp Neurol 6: 285-299.
46. Calvet J, Calvet MC, Scherrer J (1964) Etude stratigraphique corticale de l'activité EEG spontanée. Electroencephalogr. Clin Neurophysiol 17: 109-125.
47. Robinson PA, Rennie CJ, Wright JJ, Bahramali H, Gordon E, et al. (2001b) Prediction of electroencephalographic spectra from neurophysiology. Phys Rev E 63: 021903-1–021903-18.
48. Thatcher RW, North DM, Curtin RT, Walker RA, Biver CJ, et al. (2001) An EEG severity index of traumatic brain injury. J Neuropsychiatry Clin Neurosci 13(1): 77–87.
49. Gabriel S, Lau RW, Gabriel C (1996b) The dielectric properties of biological tissues: II. Measurements in the frequency range 10 Hz to 20 GHz. Phys Med Biol 41: 2251–2269.
50. Bedard C, Destexhe A (2009) Macroscopic models of local field potentials and the apparent 1/f noise in brain activity. Biophys J 96: 2589–2603.
51. Gabriel S, Lau RW, Gabriel C (1996a) The dielectric properties of biological tissues: I. Literature survey. Phys Med Biol 41: 2231–2249.
52. Gabriel S, Lau RW, Gabriel C (1996c) The dielectric properties of biological tissues: III. Parametric models for the dielectric spectrum tissues. Phys Med Biol 41: 2271–2293.
53. Bedard C, Kroger H, Destexhe A (2006) Model of low-pass filtering of local field potentials in brain tissue. Phys Rev E Stat Nonlin Soft Matter Phys 73(5 Pt 1): 051911.
54. Weiss SA, Faber DS (2010) Field effects in the CNS play functional roles. Front Neural Circuits 4: 1-10.
55. Steriade M (2001) Impact of network activities on neuronal properties in corticothalamic systems. J Neurophysiol 86: 1–39.
56. Haken H (1977) Synergetics. An Introduction. Berlin, Heidelberg, NY: Springer.
57. Bernander O, Douglas RJ, Martin KAC, Koch C (1991) Synaptic background activity influences spatiotemporal integration in single pyramidal cells. Proc Natl Acad Sci U S A 88: 11569–11573
58. Stam CJ (2005) Nonlinear dynamical analysis of EEG and MEG: review of an emerging field. Clin Neurophysiol 116: 2266–2301.
59. Wackermann J, Allefeld C (2007) On the meaning and interpretation of global descriptors of brain electrical activity. Including a reply to X. Pei et al. Int J Psychophysiol 64: 199–210.
